# Extrafield Activity Shifts the Place Field Center of Mass to Encode Aversive Experience

**DOI:** 10.1523/ENEURO.0423-17.2019

**Published:** 2019-03-22

**Authors:** Omar Mamad, Beshoy Agayby, Lars Stumpp, Richard B. Reilly, Marian Tsanov

**Affiliations:** 1 Trinity College Institute of Neuroscience; 2 School of Psychology; 3 Trinity Centre for Bioengineering; 4 School of Engineering; 5 School of Medicine, Trinity College Dublin, Dublin 2, Ireland

**Keywords:** amygdala, aversion, field plasticity, hippocampus, optogenetics, place cells

## Abstract

Hippocampal place cells are known to have a key role in encoding spatial information. Aversive stimuli, such as predator odor, evoke place field remapping and a change in preferred firing locations. However, it remains unclear how place cells use positive or negative experiences to remap. We investigated whether CA1 place cells, recorded from behaving rats, remap randomly or whether their reconfiguration depends on the perceived location of the aversive stimulus. Exposure to trimethylthiazoline (TMT; an innately aversive odor), increased the amplitude of hippocampal β oscillations in the two arms of the maze in which TMT exposure occurred. We found that a population of place cells with fields located outside the TMT arms increased their activity (extrafield spiking) in the TMT arms during the aversive episodes. Moreover, in the subsequent post-TMT recording, these cells exhibited a significant shift in their center of mass (COM) towards the TMT arms. The induction of extrafield plasticity was mediated by the basolateral amygdala complex (BLA). Photostimulation of the BLA triggered aversive behavior, synchronized hippocampal local field oscillations, and increased the extrafield spiking of the hippocampal place cells for the first 100 ms after light delivery. Optogenetic BLA activation triggered an increase in extrafield spiking activity that was correlated with the degree of place field plasticity. Furthermore, BLA-mediated increase of the extrafield activity predicts the degree of subsequent field plasticity. Our findings demonstrate that that the remapping of hippocampal place cells during aversive episodes is not random but it depends on the location of the aversive stimulus.

## Significance Statement

Here, we successfully decoded the pattern of place field reconfiguration to an aversive experience after exposure to an innately aversive odor. For the first time, we reveal that the degree of place field plasticity closely correlates with the extrafield spiking rate during aversive episodes. Using a spatial optogenetic technique in behaving rats, we show that the link between aversive behavior and place field reconfiguration is mediated by the spatially confined activation of the basolateral amygdala (BLA). These findings demonstrate that the hippocampus not only maps the spatial environment but also stores the affective value of experienced events.

## Introduction

The hippocampus stores information about spatial and nonspatial experiences (Knierim, 2003). Current theories propose that memory of spatial location is encoded by hippocampal place cells (O’Keefe and Nadel, 1978), but there is scarce information about how these neurons encode nonspatial information, such as aversive episodes. Changes in the environment are believed to evoke plasticity of the hippocampal place cell representation, a phenomenon known as remapping (Muller and Kubie, 1987; Jeffery and Hayman, 2004). It is recognized that aversion evokes place field remapping (Moita et al., 2004; Kim et al., 2015), whereby a subset of place cells in hippocampal area CA1 change their preferred firing locations in response to predator odor (Wang et al., 2012). However, it is still unclear which place cells remap in response to fearful experience and which place cells preserve their spatial fields. Here, we examined the principles governing aversion-induced place field remapping.

We hypothesized that place cell remapping depends on the spatial location of the aversive stimulus perception. To test this, we measured the change in place field center of mass (ΔCOM), which is a sensitive indicator of experience-dependent place field reconfiguration (Mehta et al., 1997; Knierim, 2002; Lee et al., 2004, 2006). Specifically, we evaluated the aversion-induced long-term shift in the ΔCOM for all place fields. We predicted that place cell remapping will depend on the spatial location of the aversive stimulus perception. This hypothesis relates to the recent finding that ΔCOM indicates rewarding contextual experience (Mamad et al., 2017). Here, we used trimethylthiazoline (TMT), a constituent of fox urine, which is an innately aversive odor to rodents (Myers and Rinaman, 2005; Kobayakawa et al., 2007). The advantage of using TMT is that it induces mild conditioning (Rosen et al., 2008). This allowed us to compare the place fields’ COM recorded from the session before and after exposure to TMT. While an electric foot-shock evokes strong and lasting aversive effect, exposure to TMT leads to short-lasting conditioning. Long-lasting aversion associated with particular place leads to path undersampling of this location where the insufficient number of passes results in incomplete formation of place fields (Hok et al., 2012), leading to imprecise evaluation of their properties (Navratilova et al., 2012).

During olfaction-driven behavior, β oscillations sustain long-range interactions between distant brain structures, including the hippocampus (Martin et al., 2007). The β frequency band (15–30 Hz) has been reported as a reliable indicator for the detection of aversive olfactory signals by the limbic circuitry (Igarashi et al., 2014). We analyzed the amplitude of hippocampal β activity to determine the section of the maze in which the aversive odor TMT was primarily perceived. The TMT protocol allows for instant association between the aversive episode and spatial location, which is easily detected by avoidance behavior and validated by β rhythm amplitude. We then investigated whether the degree of ΔCOM differed when TMT was perceived in the main place field compared to TMT perception outside the main place field. Extrafield spiking occurs outside the main place field (Huxter et al., 2003) and extrafield spikes, previously considered to be noise, are now proposed to play an essential role in information processing and learning and memory formation (Johnson and Redish, 2007; Johnson et al., 2009; Epsztein et al., 2011; Ferguson et al., 2011; Wu et al., 2017). Here, we explored whether intrafield or extrafield spikes mediate experience-dependent encoding of aversive episodes.

Hippocampal responses to aversion may occur via the amygdala; this was therefore another focus of the present study. Basolateral amygdala complex (BLA) is particularly involved in odor-evoked fear conditioning (Wallace and Rosen, 2001; Anderson et al., 2003; de Araujo et al., 2003). The amygdala plays a key role in aversion-associated memory behavior, its activation induces place field remapping, whereas amygdalar inhibition or lesioning prevent this remapping (Donzis et al., 2013; Kim et al., 2015). Furthermore, electrical stimulation of BLA decreases the stability of CA1 place fields (Kim et al., 2012). Even so, the mechanisms underlying these effects have yet to be investigated. To understand the mechanism of aversion-induced place field remapping, we optogenetically activated pyramidal neurons from the BLA. We hypothesized that optogenetic stimulation of BLA will induce different degree of remapping when paired with the intrafield spiking compared to stimulation paired with the extrafield spiking. We photostimulated the BLA to compare the patterns of ensemble reconfiguration after (1) aversion-induced field remapping, and (2) optogenetic BLA activation. We examined whether the place cells remap randomly or according to the location of the aversive stimulus.

## Materials and Methods

### Ethics statement

We conducted our experiments in accordance with directive 2010/63/EU of the European Parliament and of the council of 22 September 2010 on the protection of animals used for scientific purposes and the S.I. No. 543 of 2012, and followed Bioresources Ethics Committee (individual authorization number AE19136/I037; procedure numbers 230113-1001, 230113-1002, 230113-1003, 230113-1004, and 230113-1005) and international guidelines of good practice (project authorization number: AE19136/P003).

### Animals

Male, three to six months old, Lister-Hooded rats (RGD catalog #2312466, RRID:RGD_2312466) were individually housed for at least 7 d before all experiments, under a 12/12 h light/dark cycle, provided with water *ad libitum*. Prior the experiments restricted feeding diet kept the rats on 80% of their expected weight when fed *ad libitum*. Experiments were performed during the light phase.

### Surgical implantation of recording electrodes and recording techniques

Eight tetrodes and optic fiber were implanted in hippocampal CA1 area: –3.8 AP, 2.3 ML, and 1.8 mm dorsoventral to dura. The optic fiber and tetrodes were implanted unilaterally in BLA: 2.4 AP, 4.9 ML, and 7.0 mm dorsoventral to dura. The recordings were performed as previously described (Mamad et al., 2017). After a minimum one-week recovery, subjects were connected, via a 32-channel headstage (Axona Ltd.) to a recording system, which allowed simultaneous animal position tracking. Signals were amplified (10,000- to 30,000-fold) and band-pass filtered between 380 Hz and 6 kHz for single-unit detection. To maximize cell separation, only waveforms of sufficient amplitude (at least three times the noise threshold) were recorded. Candidate waveforms were discriminated off-line using graphical software Tint (BatchTINTV2, RRID:SCR_014804), which allows waveform separation based on multiple features including spike amplitude, spike duration, maximum and minimum spike voltage, and the time of occurrence of maximum and minimum spike voltages. Autocorrelation histograms were calculated for each unit, and the unit was removed from further analysis if the histogram presented spiking within the first 1 ms (refractory period), inconsistent with good unit isolation. Only stable recordings across consecutive days were further analyzed. The stability of the signal was evaluated by the cross-correlation of spike amplitudes and similarity comparison of the spike clusters between the sessions. Electrode stability was assessed off-line by comparison of waveforms and cluster distributions. The single unit signals from the last recording session and the probe were compared for waveform similarity, cluster location, size, and boundaries. Peak and trough amplitudes of the averaged spike waveforms were compared using Pearson’s *r*. Values for *r* ≥ 0.8 indicated that the same populations of cells were recorded throughout the last recording session and the probe.

### Hippocampal unit identification and spatial firing analysis

Single hippocampal pyramidal cells and interneurons were identified using spike shape and firing frequency characteristics (Ranck, 1973; Wilson and McNaughton, 1993). Firing rate maps allow for visual inspection of neurons preferred areas of firing (i.e., place fields). They were constructed by normalizing the number of spikes which occurred in specific pixelated coordinates by the total trial time the animal spent in that pixel. This produced maps depicting the place fields of each cell. Maps were quantified in Hz (smoothed maps). We defined place field size as the region of the arena in which the firing rate of the place cell was greater than 20% of the maximum firing frequency (Brun et al., 2002). Appearance of sharp waves and ripples during immobility, triggers the spiking of multiple place cells (Wu et al., 2017). To avoid spikes reactivation during sharp wave ripple we excluded spikes that occurred during epochs with running speeds below 5 cm/s (Alme et al., 2014; Grosmark and Buzsaki, 2016). The place field analysis included only epochs during which the animal’s velocity was at least 5 cm/s.

### Extrafield spiking

Place fields were defined as areas of nine contiguous pixels (2.5 cm^2^/pixel) with average activity >20% of the field maximum rate. Extrafield spiking was defined as spikes occurring outside of the identified place field areas (Huxter et al., 2003; Johnson and Redish, 2007). The extrafield spiking thus included secondary place fields with sizes smaller than nine contiguous pixels or with averaged firing rate smaller than 20% of the maximum firing rate (Brun et al., 2002; Huxter et al., 2003; Johnson and Redish, 2007).

### Measurement of local field activity

The local field potential (LFP) was sampled at 250 Hz and stored for further off-line analysis. LFP signal frequency analysis was carried out using MATLAB’s Signal Processing Toolbox (MATLAB, RRID:SCR_001622) where the power was calculated using the short-time Fourier transform of the signal (Hanning window of 2 s, with overlap of 1 s) and interpolated into color-coded power spectrograms. Information was displayed as the magnitude of the time-dependent Fourier Transform versus time in a color gradient graph with the maximum corresponding to 0 dB.

### Phase-locking value

To evaluate the effect of optogenetic BLA stimulation we compared the hippocampal local field oscillations of a single electrode between multiple trials (Mamad et al., 2015). Phase-locking statistics measures the significance of the phase covariance between separate signals and allows direct quantification of frequency-specific synchronization (i.e., transient phase-locking) between LFPs (Lachaux et al., 1999). The phase-locking value is the amplitude of the first circular moment of the measured phase difference between two phases (Lachaux et al., 1999; Canolty et al., 2010). The phase-locking value ranges between 0 and 1; 0 signifying purely random rise and fall whereas a value of 1 signifies that one signal perfectly follows the other. To distinguish between noise-related fluctuations of the phase-locking values we compared the observed data with shuffled data (Mamad et al., 2015).

### Experimental design

The animals were trained to navigate between the northwest and southeast corners of rectangular-shaped linear track, where two pellets were continuously positioned. The animals were allowed to freely navigate in both clockwise and counter-clockwise directions of this rectangular-shaped linear track (10-cm width, 85-cm length of the arms): via the southwest (SW) arms and via the northeast (NE) arms. For the TMT experiments one of the filter papers of the track was scented with 50-µl 10% TMT (Contech). The advantage of TMT is the absence of learning curve required for associative fear conditioning. The experimental protocol involving one TMT session with duration of 12 min was designed to evoke long-lasting (>24 h) but weak place aversion response during the post-TMT recording session. The place aversion was measured only in the first 60 s of the post-TMT recording session. In the subsequent 11 min of the post-TMT recording session the animals displayed regular navigation in the TMT arms. This protocol allowed for sufficient number of passes, preventing an undersampling path measurement of the post-TMT navigation. During the TMT sessions the TMT filter papers were applied in all locations of the TMT arms for different rats; this protocol was designed to match the ChR2 protocol where the blue light was applied across all locations of the ChR2 arms. For the optic stimulation sessions, the laser was switched on when the animal entered the south arm or the west arm with continuous photostimulation trains (473 nm, 50 Hz, 5-ms pulse duration, 12 pulses per train, 0.5-s intertrain interval) until the animal exited this section of the track. The blue laser was synchronized with the video-tracking and with the recording system through hardware and DACQBASIC scripts (Axona, Ltd). The duration of each session (baseline, TMT, ChR2) was 12 min. The TMT zone (ChR2 zone) included the TMT arms (ChR2 arms) and the feeding corners. For control experiments we used filter papers of the track scented with 50-µl 10% ethanol, which was a familiar odor to the rats. The animals were habituated prior the recording sessions to the scent of ethanol.

### Clockwise and counter-clockwise place field analyses

The rat’s direction of movement was calculated for each tracker sample from the projection of the relative position of the LEDs onto the horizontal plane. The momentary angular displacement was calculated as the difference in the animal’s position between successive 50 Hz time samples. The direction time series was first smoothed by calculating a five-point running average. After smoothing, the instantaneous direction of movement was calculated as the angular displacement between successive points per time (Taube, 1995). To restrict the influence of inhomogeneous sampling on directional tuning, we separated the directionality for the pre-TMT(ChR2) and post pre-TMT(ChR2) where the animals exhibited consistent navigation, but not for the TMT(ChR2) sessions where the direction of animal’s navigation was highly inconsistent due to the aversive episode. For the linear color-coded representation of the unidirectional place fields the firing rate was normalized for each cell to the cell’s baseline maximal firing rate. The unidirectional clockwise/counter-clockwise place fields were defined as areas of nine contiguous pixels (2.5 cm^2^/pixel) with average activity >10% of the field maximum rate. Although the reduction of the place field firing rate cut-off to 10% increases the extrafield noise this approach also preserves the peripheral spiking activity considered outside the place field with the 20% cut-off approach. The firing rate difference between the pre-TMT(ChR2) and post-TMT(ChR2) recordings was normalized by the ratio of the difference over the sum of the pre-spiking and post-spiking count (see spike ratios).

### Optogenetic tools

AAV-CaMKIIa-hChR2(H134R)-eYFP-WPRE-hGH viral construct was serotyped with AAV5 coat proteins and packaged by Vector Core at the University of North Carolina with viral titers ranged from 1.5–8 × 10^12^ particles/ml. For control experiments we used virus bearing only the yellow fluorescent protein (YFP) reporter. Randomization of group allocation (ChR2 vs YFP controls) was performed using an online randomization algorithm (http://www.randomization.com/). The virus injection was applied unilaterally in the BLA (2.4 AP, 4.9 ML), with volume of 2 µl injected on two levels: 1 µl at 6.5 mm and 1 µl at 7.5 mm dorsoventral to the dura. Subsequently an optical fiber (200-μm core diameter, Thorlabs, Inc.) was chronically inserted (2.4 AP, 4.9 ML, 6.5 DV). Simultaneous optical stimulation and extracellular recording from CA1 were performed in freely-behaving rats three weeks after the surgery. The light power was controlled to be 10–15 mW at the fiber tip. Square-wave pulses with duration of 5 ms were delivered at frequency of 50 Hz.

### Spiking ratios

$$TMT\,\;spiking\,\;ratio=\frac{firing\;rate\;from\;the\;preTMT\;session\;in\;the\;TMT\;arms}{firing\;rate\;from\;the\;TMT\;session\;in\;the\;TMT\;arms}$$

$$NonTMT\,\;SR=\frac{firing\,\;rate\,\;from\,\;the\,\;preTMT\,\;session\,\;in\,\;the\,\;non\,\;TMT\,\;zone}{firing\,\;rate\,\;from\,\;the\,\;TMT\,\;session\,\;in\,\;the\,\;non\,\;TMT\,\;zone}$$

$$TMT/anti\,\;TMT\,\;passes\,\;ratio=\frac{Number\;of\,\;passes\,\;in\,\;the\,\;TMT\,\;arms}{Number\,\;of\,\;passes\,\;in\,\;the\,\;non\,\;TMT\,\;arms}$$

$$ChR2\,\;spiking\,\;ratio=\frac{firing\,\;rate\,\;from\;the\,\;preChR2\,\;session\,\;in\,\;the\,\;ChR2\,\;arms}{firing\,\;rate\,\;from\,\;the\,\;ChR2\,\;session\,\;in\,\;the\,\;ChR2\,\;arms}$$

$$NonChR2\,\;\mathrm{spiking}\;\mathrm{ratio}=\frac{firing\,\;rate\,\;from\,\;the\,\;preChR2\,\;session\,\;in\,\;the\,\;nonChR2\,\;zone}{firing\,\;rate\,\;from\,\;the\,\;ChR2\,\;session\,\;in\,\;the\,\;nonChR2\,\;zone}$$

$$ChR2/non\,\;ChR2\,\;passes\,\;ratio=\frac{Number\,\;of\,\;passes\,\;in\,\;the\,\;ChR2\,\;arms}{Number\,\;of\,\;passes\,\;in\,\;the\,\;non\,\;ChR2\,\;arms}$$

$$Pre/post\,\;normalized\,\;spikes\,\;count=\frac{\left(spikes\,\;count\,\;pre-spikes\,\;count\,\;post\right)}{\left(spikes\,\;count\,\;pre+spikes\,\;count\,\;post\right)}$$

### COM

The COM was calculated by taking the *x* and *y* averages for the rows and columns of the rate map weighted for firing rate. For the unidirectional place field analyses COM included only the spikes located in the place field defined by the field area smaller than 10% of the maximum firing rate, while for the bidirectional analyses we used all spikes with 20% cut-off. The spatial position of the place cell was defined for all recorded spikes as the COM of the firing rate distribution within the maze coordinates. The COM of the place cells’ spike distribution is calculated as follows:

$${COM}_{x}=\frac{\sum_{i=1}^{N_{x}}\sum_{j=1}^{N_{y}}f_{ij}\cdot i}{\sum_{i=1}^{N_{x}}\sum_{j=1}^{N_{y}}f_{ij}}\cdot l_{bin}-\frac{l_{bin}}{2}$$

$${COM}_{y}=\frac{\sum_{i=1}^{N_{x}}\sum_{j=1}^{N_{y}}f_{ij}\cdot j}{\sum_{i=1}^{N_{x}}\sum_{j=1}^{N_{y}}f_{ij}}\cdot l_{bin}-\frac{l_{bin}}{2}$$

Where *N*_x_, *N*_y_ define the number of bins in the arena in *x*-, *y*-direction; *f*_i,j_ is firing frequency in bin *i*, *j*; *l*_bin_ is the bin size.

Given the origin O (O_x_,O_y_), which denotes the northwest (NW) corner of the Cartesian coordinate system, and the direction of the symmetry axis D (D_x_,D_y_), which denotes the line between the SW and NE corners, the distance of the COM (COM_x_,COM_y_) to the symmetry axis is calculated as follows:

$${dist}_{COM/sym}=\frac{\left|det\left[\begin{array}{cc} D_{x}-O_{x} & D_{y}-O_{y}\\ {COM}_{x}-O_{x} & {COM}_{y}-O_{y} \end{array}\right]\right|}{\sqrt{{\left(P_{x}-O_{x}\right)}^{2}+{\left(D_{y}-O_{y}\right)}^{2}}}$$

Where *P* is the shortest distance between the COM and the symmetry line. For the rectangular-shaped linear track, the arena borders are defined as the square surrounding all motion tracking sample points with an equal distance to the real limits of the arena at all sides.

Using *dist*_COM_, we calculate the distance between O and P as follows:

$$\overset{¯}{OP}=\sqrt{{{(COM}_{x}}^{2}+{{COM}_{y}}^{2})-{{dist}_{COM}}^{2}}$$

The COM distance normalized by the arena width perpendicular to the symmetry axis through the COM is calculated as:

$${dist}_{norm}=\left\{\begin{array}{c} \frac{{dist}_{COM}}{\overset{¯}{OP}\cdot C},\overset{¯}{OP}<\frac{\overset{¯}{OM}}{2}\\ \frac{{dist}_{COM}}{\left(\overset{¯}{OM}-\overset{¯}{OP}\right)\cdot C},\overset{-}{OP}>\frac{\overset{¯}{OM}}{2} \end{array}\right\}$$

Where $\overset{-}{OM}$ is the diagonal of a square enclosing all motion tracking data points, and *C* is a motion tracking data factor and, in this case, set to 0.85 for the linear rectangular track.

### COM angle (COMa)

The COMa computes the shift of place field COM towards the TMT arms using radial direction in degrees where the axis between the feeding zones denotes 45°. COMa is calculated as follows:

$$\theta_{COM}=\left\{\begin{array}{c} 45{}^{\circ}\cdot \left(1-{dist}_{norm}\right),{COM}_{x}<{COM}_{y}\\ 45{}^{\circ}\cdot \left(1+{dist}_{norm}\right),{COM}_{x}>{COM}_{y} \end{array}\right\}$$

Where *θ_COM_*is COMa; COM_x_, COM_y_: *x*-, *y*-coordinate of COM, *dist_norm_* normalized COM distance. The shift in the COM (ΔCOM) is the absolute difference between the pre-TMT(ChR2) and post-TMT(ChR2) recordings:

|ΔCOM| = COM_(pre-TMT)_ – COM_(post-TMT)_

|ΔCOM| = COM_(pre-ChR2)_ – COM_(post-ChR2)_

All algorithms were implemented in MATLAB.

### Histology

At the end of the study, brains were removed for histological verification of electrode localization. Rats were deeply anesthetized with sodium pentobarbital (390 mg/kg) and perfused transcardially with ice-cold 0.9% saline followed by 4% paraformaldehyde. Brains were removed, post-fixed in paraformaldehyde for up to 24 h and cryoprotected in 25% sucrose for >48 h. Brains were sectioned coronally at 40 µm on a freezing microtome. Primary antibody incubations were performed overnight at 4°C in PBS with BSA and Triton X-100 (each 0.2%). The concentration for primary antibodies was anti-CamKIIα 1:500 (Millipore, catalog #MAB8699, RRID:AB_2067919). Sections were then washed and incubated in PBS for 10 min and secondary antibodies were added (1:500) conjugated to Alexa Fluor 594 dye from Invitrogen (Molecular Probes catalog #A-11032, RRID:AB_141672) for 2 h at room temperature.

For visualization, the sections were mounted onto microscope slides in phosphate-buffered water and cover-slipped with Vectashield mounting medium (Vector Laboratories catalog #H-1200, RRID:AB_2336790). The YFP fluorescence was evaluated within a selected region that was placed below the fiber tip in an area of 1.5 × 1.5 mm. Fluorescence was quantified based on the average pixel intensity within the selected region (Witten et al., 2011). The stained sections were examined with an Olympus IX81 confocal microscope at 594 nm for Alexa Fluor secondary antibody and 488 nm for ChR2-YFP. CamKIIα-positive neurons were identified based on the expression of red fluorescence, whereas ChR2-positive neurons were identified by the expression of green fluorescence. Co-localization of Alexa Fluor 594 and YFP was determined manually using ImageJ software (RRID:SCR_003070).

### Statistical analysis

Two different approaches were used to calculate the sample size (Karalis et al., 2016). We performed power analyses to establish the required number of animals for experiments in which we had sufficient data on response variables. For experiments in which the outcome of the intervention could not be predetermined, we employed a sequential stopping rule. This approach allows null-hypothesis tests to be used subsequently by analyzing the data at different experimental stages using *t* tests against Type II error. The experiment was initiated with four animals per group; if *p* < 0.05, the testing was continued with two or three more animals to increase statistical power. In the case of *p* > 0.36, the experiment was discontinued and the null hypothesis was accepted (Karalis et al., 2016). All data were analyzed using SPSS Software. Statistical significance (Table 1) was estimated by using a two-tailed independent samples *t* test for non-paired data or a paired samples Student *t* test for paired data. Repeated measures were evaluated with one-way or two-way ANOVA paired with *post hoc* Bonferroni test. Correlations between data sets were determined using Pearson’s correlation coefficient. The probability level interpreted as significant was fixed at *p* < 0.05. Data are plotted as mean ± SEM.

**Table 1.** Statistical table

Line location	Data structure	Type of test	Power
a	Normal distribution	Two-tailed paired *t* test	0.95
b	Normal distribution	One-way ANOVA	0.95
c	Normal distribution	Two-way ANOVA with Bonferroni *post hoc* test	0.95
d	Normal distribution	Two-tailed unpaired *t* test	0.95
e	Normal distribution	Pearson’s correlation	0.95
f	Normal distribution	One-way ANOVA with Bonferroni *post hoc* test	0.95

### Data availability

Dataset of all experimental files is available at Figshare public repository. URL: https://doi.org/10.6084/m9.figshare.5336026.v1.

## Results

### Increase in hippocampal β amplitude is associated with TMT-mediated aversion

**Figure 1.**
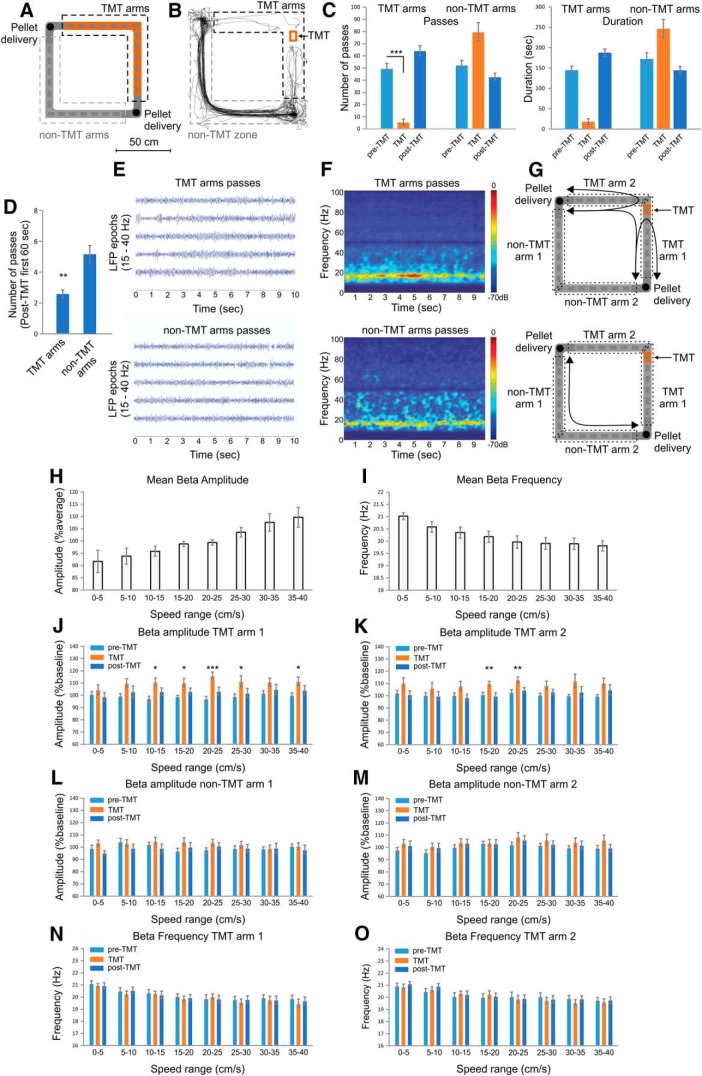
Increase in hippocampal β activity during odor-triggered place avoidance. ***A***, Setup of the rectangular-shaped linear track. Locations of the TMT are marked with red in the NE arms of the track. The black dashed line indicates the TMT arms, while grey dashed line marks the non-TMT arms. To match the TMT protocol with the subsequent ChR2 photostimulation protocol, we applied TMT scent papers to different locations in both TMT arms. ***B***, Representative navigation path of a rat during the TMT session. Note the place avoidance of the TMT arms. ***C***, Number of passes (left graph) and duration in seconds (right graph) in the TMT and non-TMT arms during the pre-TMT, TMT, and post-TMT sessions; ***p < 0.001. Error bars, mean ± SEM. ***D***, Number of passes through the TMT and non-TMT arms for the first 60 s of the post-TMT session; **p < 0.01. ***E***, Representative bandpass-filtered (15–40 Hz) LFP traces recorded during the passes in the TMT arms (top panel) and in the non-TMT arms (bottom panel). Time 0 indicates the start of the path trajectory from the pellet delivery location. ***F***, Representative averaged bandpass-filtered (15–40 Hz) color-coded power spectrogram of all passes in the TMT arms (top panel) and in the non-TMT arms (bottom panel). The averaged power spectrogram includes the variability of pass duration and speed during the navigation across the arms. ***G***, Spatial dissociation of odor perception for TMT arms (top panel) and non-TMT arms (bottom panel). The arm with TMT scent paper was named TMT arm 1. The adjacent arm, part of the same food-navigation loop, was TMT arm 2. The opposite of TMT arm 1 was named non-TMT arm 1, while the opposite of TMT arm 2 was non-TMT arm 2. The scent was detected by the animal at different locations during the navigation passes across the TMT arms. Black arrows indicate the possible path trajectories of the animals. Mean β amplitude (***H***) and mean β frequency (***I***) measured for rats with a whole-body speed of 0–40 cm/s, in bins of 5 cm/s. ***J***, β Amplitude for TMT arm 1 and ***K*** TMT arm 2 (right) as percentages of the pre-TMT session values for the entire track; ***p < 0.001, **p < 0.01, *p < 0.05. Error bars, mean ± SEM. β Amplitude for non-TMT arm 1 (***L***) and non-TMT arm 2 (***M***) as percent of the pre-TMT session values for the entire track. The amplitude values are presented as a function of the animal’s whole-body speed, where β amplitude is evaluated for a speed range of 0–40 cm/s in bins of 5 cm/s. Error bars, mean ± SEM. β Frequency for TMT arm 1 (***N***) and TMT arm 2 (***O***) for speed range of 0–40 cm/s in bins of 5 cm/s. Error bars, mean ± SEM.

To evoke aversion episodes in rats chronically implanted with tetrodes in the hippocampal CA1 region, we applied 10% TMT to a small area (10 × 15 cm) on a rectangular-shaped linear track (Fig. 1*A*). The animals were allowed to freely navigate in both clockwise and counter-clockwise directions between the northwest and southeast corners of the track, where two pellets were continuously positioned. The experimental design consisted of three recording sessions (12 min each) conducted within a total period of 48 h. The three sessions comprised baseline recording (pre-TMT), a subsequent recording during which TMT was located in one of the arms of the track (TMT), and a final recording without TMT (post-TMT).

During the TMT session, the rats (*n* = 12) avoided navigating across the TMT-scented section of the track (Fig. 1*B*; Movie 1), demonstrating place avoidance, expressed by reduced number of passes compared to the pre-TMT session (*t* test, *n* = 12 rats, *t*_(11)_ = 10.4, *p* < 0.001^a^; Fig. 1*C*). The TMT session was designed to evoke a brief aversive response in the post-TMT recording session, and subsequent extinction, to avoid navigation undersampling. The path sampling data of the pre-TMT and post-TMT sessions showed sufficient dwell time in both TMT and non-TMT arms with sufficient path sampling for all rats (Fig. 1*C*; Table 2). The association of the TMT arms with the aversive odor was evident during the first minute of navigation in the post-TMT session (*t* test, *n* = 12, *t*_(11)_ = –4.244, *p* = 0.001^a^; Fig. 1*D*), whereby the average number of passes (2.58 ± 0.3) was significantly lower for the TMT arms than for the non-TMT arms (5.16 ± 0.7). This result validated the application of TMT as experimental protocol for the induction of mild aversion.

**Table 2.** Path sampling from recordings with aversive experimental design

TMT-NE rat number	Pre-TMT session				TMT-NE session				Post-TMT session			
	Passes		Duration (s)		Passes		Duration (s)		Passes		Duration (s)	
	SW	NE	SW	NE	SW	NE	SW	NE	SW	NE	SW	NE
Rat 1	38	42	102.2	112.7	50	0	150.2	0	42	31	134.6	107.5
Rat 2	33	41	156.9	181.4	91	9	266.4	65.4	60	51	187.5	160.7
Rat 3	31	48	122.8	218.8	62	0	229.4	0	74	24	241.0	91.92
Rat 4	53	59	153.1	183.1	70	0	272.2	0	47	43	189.5	154.9
Rat 5	48	54	150.4	194.3	78	0	260.7	0	75	37	233.2	116.5
Rat 6	64	80	163.1	210.9	101	0	308.8	0	72	60	194.2	189.9
TMT-SW rat number	Pre-TMT session				TMT-SW session				Post-TMT session			
	Passes		Duration (s)		Passes		Duration (s)		Passes		Duration (s)	
	SW	NE	SW	NE	SW	NE	SW	NE	SW	NE	SW	NE
Rat 7	31	50	105.8	157.9	15	47	50.9	187.9	38	42	112.0	153.0
Rat 8	56	59	170.8	152.5	0	95	0	350.3	41	63	136.5	166.9
Rat 9	40	39	160.7	107.1	0	106	0	258.7	49	87	152.6	159.4
Rat 10	61	51	167.7	132.7	16	46	30.2	79.2	44	73	154.2	180.6
Rat 11	60	52	175.5	138.4	24	82	52.7	248.3	42	65	163.2	188.4
Rat 12	59	80	186.1	198.6	5	130	17.1	346.1	55	72	190.9	224
ChR2-SW rat number	Pre-ChR2 session				ChR2-SW session				Post- ChR2 session			
	Passes		Duration (s)		Passes		Duration (s)		Passes		Duration (s)	
	SW	NE	SW	NE	SW	NE	SW	NE	SW	NE	SW	NE
Rat 1	46	34	140.7	105.2	27	49	175.4	119.5	29	13	88.0	181.2
Rat 2	31	54	98.4	153.9	23	52	75.4	159.8	36	54	166.1	155.4
Rat 3	26	46	84.7	102.2	21	41	84.3	160.6	31	54	107.8	167.1
Rat 4	35	41	118.3	131.0	11	38	146.1	167.0	45	47	170.1	135.8
Rat 5	33	48	100.3	130.7	15	38	101.6	128.4	47	44	113.6	96.0
Rat 6	36	42	114.6	130.5	13	40	201.7	197.4	38	40	169.7	151.5

Number of passes and dwell time (duration in seconds) measured in the SW and NE arms for TMT and ChR2 groups of animals during the pre-TMT(ChR2), TMT(ChR2), and post-TMT(ChR2) recording sessions. The rats exposed to TMT include TMT-NE, and TMT-SW groups.

Movie 1. **TMT-induced aversion.** Navigation on a rectangular linear track by a rat with chronically implanted tetrodes in the hippocampal CA1 region. The animal was trained to navigate between the SW and NE corners, where two pellets were continuously positioned. TMT was placed in a particular location of the track, which is denoted in red. 10.1523/ENEURO.0423-17.2019.movie.1

As hippocampal oscillations in the β frequency range (15–30 Hz) indicate successful odor discrimination (Igarashi et al., 2014), we considered the increase in β amplitude as an indicator of aversive odor processing in the hippocampal network. To identify whether the odor discrimination was restricted to the TMT section of the maze or whether it occurred over the entire track, we analyzed the amplitude of the hippocampal β frequency band (representative data are shown in Fig. 1*E*,*F*) for each arm of the track (Fig. 1*G*). The hippocampal β amplitude (ANOVA, *n* = 12, *F*_(1,7)_ = 3.1, *p* = 0.005^b^; Fig. 1*H*) and frequency (ANOVA, *n* = 12, *F*_(1,7)_ = 3.8, *p* = 0.002^b^; Fig. 1*I*) were associated with the animal’s whole-body linear speed, where β amplitude consistently increased and β frequency consistently decreased as speed increased. Concurrently, the TMT-induced aversion diminished the animals’ linear speed (Movie 1). To avoid any biasing effect of speed on β parameters, we analyzed the effect of TMT on β oscillations for different speed bands. The highest increase in β amplitude was evident during the passes through the arm with TMT odor (TMT arm 1, ANOVA, *n* = 12, *F*_(2,10)_ = 17.5, *p* < 0.001^c^; Fig. 1*J*), with a significant increase for five speed ranges. A significant β amplitude increase was also present in the arm adjacent to the TMT arm (TMT arm 2, ANOVA, *F*_(2,10)_ = 24.3, *p* < 0.001^c^; Fig. 1*K*). The opposite two arms, non-TMT arm 1 and non-TMT arm 2, were characterized by nonsignificant changes in β amplitude (ANOVA, between groups, *n* = 12; non-TMT arm 1: *F*_(2,10)_ = 3.0, *p* = 0.094^c^; non-TMT arm 2: *F*_(2,10)_ = 1.3, *p* = 0.295^c^ Fig. 1*L*,*M*). We found no significant effect of TMT on the frequency of β oscillations (ANOVA, TMT arm 1: *n* = 12, *F*_(2,10)_ = 0.7, *p* = 0.493^c^; TMT arm 2: *n* = 12, *F*_(2,10)_ = 1.2, *p* = 0.349^c^; non-TMT arm 1: *n* = 12, *F*_(2,10)_ = 2.1, *p* = 0.164^c^; non-TMT arm 2: *n* = 12, *F*_(2,10)_ = 1.8, *p* = 0.213^c^; Fig. 1*N*,*O*). These results indicate that the effect of TMT on the hippocampal local field activity was restricted to the two TMT arms.

### Place cell activity in the TMT arms during the TMT session was correlated with the degree of field reconfiguration

**Figure 2.**
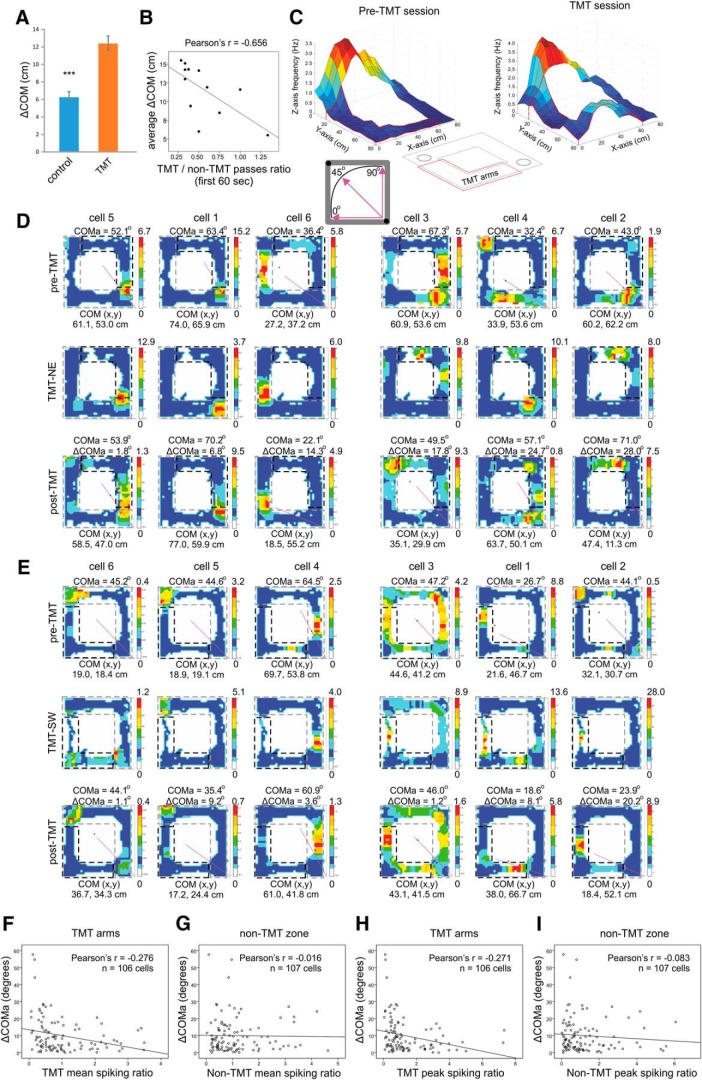
Individual place field reconfiguration after exposure to TMT. ***A***, COM shift (ΔCOM) of place cells recorded from a control group of rats exposed to familiar odor (10% ethanol) and group of rats exposed to innately aversive odor (10% TMT); ***p < 0.001. Error bars, mean ± SEM. ***B***, Correlation between the average ΔCOM for each rat and the ratio of the TMT- over the non-TMT arms passes for the first 60 s of the post-TMT session. ***C***, left, Color-coded 3D-spatial map of a sample place cell with place field located outside the TMT arms (the TMT arms are marked with a red line), recorded during pre-TMT session. The *x*- and *y*-axes represent the coordinates of the rectangular track, while the *z*-axis and color scale represent the firing rate of the recorded neuron. Right, As left, but recorded during the TMT session. The inset below shows the position of the TMT arms with a red line (circles indicate the pellet delivery corners). The spiking TMT ratio represents the mean (peak) firing rate of the pre-TMT session over the TMT session. The inset below shows the radial representation of COMa. The straight purple line indicates the COM position with respect to the main axis of the track between the food zones (black dots). A value of 45° indicates a COM that is evenly distributed across the main axis of the track, 0° indicates COM fully distributed within the SW section and 90°, in the NE section of the track. ***D***, Six sample place cells recorded from rat of the NE group during pre-TMT session (upper panels), TMT session (middle panels), and post-TMT session (lower panels). The straight purple line denotes the COMa for each cell between SW at 0° and NE at 90°. Note that some of the place cells exhibited a higher firing rate in the TMT arms during the TMT sessions (panels positioned on the right half) compared to place cells with little or no spiking (panels positioned on the left half). ***E***, Six sample place cells recorded from rat of the SW group during pre-TMT session (upper panels), TMT session (middle panels), and post-TMT session (lower panels). ***F***, Correlation between ΔCOMa and TMT spiking ratio based on the mean spiking rate of the place cells in the TMT arms. ***G***, Correlation between ΔCOMa and non-TMT spiking ratio on the mean spiking rate of the place cells in the non-TMT zone. ***H***, As for F, but with peak TMT spiking ratio instead of mean TMT spiking ratio. ***I***, As for ***G***, but with peak TMT spiking ratio instead of mean TMT spiking ratio.

We next explored whether TMT exposure increased the remapping propensity of the place cells, measured as ΔCOM for all spikes. ΔCOM is an efficient approach for evaluating field plasticity because this parameter represents spiking as a function of occupancy for each pixel and detects both spatial and rate remapping (Knierim, 2002; Lee et al., 2004). We defined ΔCOM as a shift of the COM between the pre-TMT and post-TMT recordings. The average ΔCOM for the place cells from the TMT group was 12.44 ± 0.8 cm (Fig. 2*A*). This was significantly greater than the average ΔCOM from control rats subjected to the hedonically neutral odor (6.32 ± 0.6 cm with 10% ethanol; *t* test, control group *n* = 57, TMT group *n* = 106, *t*_(161)_ = 5.2, *p* < 0.001^d^). Furthermore, there was a significant negative correlation between the ratio of TMT to non-TMT passes for the first 60 s and the magnitude of ΔCOM (*r* = –0.656, *n* = 12, *p* = 0.020^e^; Fig. 2*B*). This result established the link between ΔCOM and TMT-induced aversive experience.

**Figure 3.**
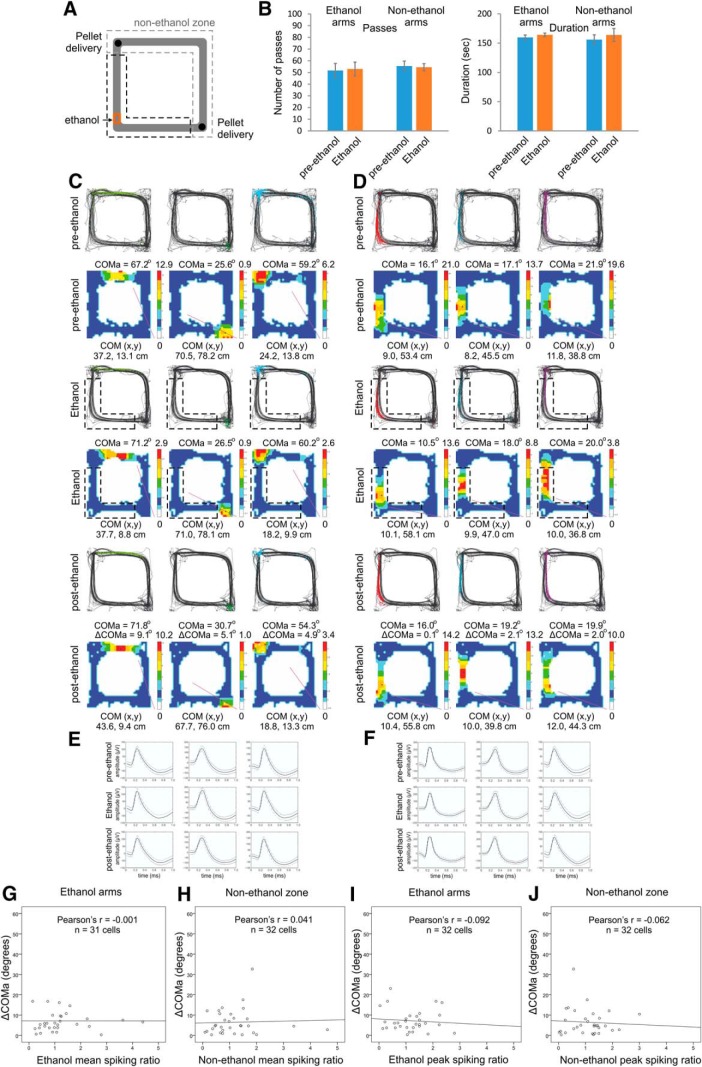
Place field stability in control conditions. ***A***, Schematic representation of the application of a familiar scent, ethanol (marked with red) in the SW arms of rectangular-shaped linear track for the control group of animals. ***B***, Number of passes (left panel) and duration in seconds (right panel) counted in the ethanol arms (left) and non-ethanol arms (right) for the control group of rats, before (blue) and during (red) the odor exposure. Error bars, mean ± SEM. ***C***, Three place fields from a representative rat with fields located outside the ethanol arms during pre-ethanol (top), ethanol (middle), and post-ethanol (bottom) sessions. For each session, the upper panels show the animal trajectory with spikes, marked with colored dots, while the lower panels show color-coded firing rate. ***D***, Three place fields from the same animal with fields located inside the ethanol arms during pre-ethanol (top), ethanol (middle), and post-ethanol (bottom) sessions. For each session, the upper panels show the animal trajectory with spikes, marked with colored dots, while the lower panels show color-coded firing rate. ***E***, Waveforms of the place cells shown in ***C***. ***F***, Waveforms of the cells shown in ***D***. The solid line shows the average waveform shape; the dashed lines show the 1 SD confidence intervals. ***G***, Correlation between ΔCOMa and the ethanol spiking ratio based on the mean firing rate of the place cells’ spikes in the ethanol arms. ***H***, Correlation between ΔCOMa and the ethanol spiking ratio based on the mean firing rate of the place cells’ spikes in the nonethanol zone. ***I***, As for ***G***, but with peak ethanol spiking ratio instead of mean spiking ratio. ***J*,** As for ***H***, but with peak ethanol spiking ratio instead of mean spiking ratio.

Next, we investigated whether the spiking activity of individual place cells during the TMT episodes (Fig. 2*C*) was related to the magnitude of the subsequent ΔCOM. We used radial and linear approaches to investigate ΔCOM. The radial approach measures the shift in each place cell’s COMa (ΔCOMa). ΔCOMa estimates the radial shift of the COM between the pre-TMT and post-TMT sessions, across the main axis of the track that extends between the food corners (Fig. 2*C*, inset), where SW corner denotes 0° and NE corner denotes 90° (see Methods and Materials). We evaluated ΔCOMa for group of animals where TMT was positioned in the north or east arms (NE group; Fig. 2*D*) and for group of animals where TMT was positioned in south or west arms of the track (SW group; Fig. 2*E*). To establish the link between the change of place cell’s spiking and subsequent place field plasticity we correlated ΔCOMa with the TMT-associated change in the place cell firing rate. The TMT-associated change in firing rate (TMT spiking ratio) was calculated as the ratio of pre-TMT to TMT firing rates from the recorded spikes. We found a significant negative correlation between ΔCOMa and the TMT spiking ratio of the mean firing rate measured for the TMT arms (*r* = –0.276, *p* = 0.004^e^, *n* = 106; Fig. 2*F*), but there was no significant correlation between same two variables for the non-TMT zone (Fig. 2*G*; Table 3). Similarly, the correlation between ΔCOMa and the spiking ratio of the peak firing rate was significant for the TMT arms (Fig. 2*H*; Table 3) but not for the non-TMT zone (Fig. 2*I*; Table 3). By contrast, in control rats (Fig. 3*A*,*B*), we found no significant correlation between ΔCOMa (Fig. 3*C*,*D*) and the mean spiking ratio for either the ethanol arms (Fig. 3*G*; Table 3) or the non-ethanol zone (Fig. 3*H*; Table 3). The correlations between ΔCOMa and peak spiking ratios were also non-significant (Fig. 3*I*,*J*; Table 3). These results demonstrate that place field remapping correlates to the change in place cell’s spiking during the preceding aversive episode.

**Table 3.** Correlation between spiking ratios and ΔCOMa

Spiking ratio	*r*	*p* value	*n*
Mean TMT spiking ratio for TMT arms	–0.276	0.004^e^	106
Mean TMT spiking ratio for non-TMT zone	–0.016	0.871^e^	107
Peak TMT spiking ratio for TMT arms	–0.271	0.005^e^	106
Peak TMT spiking ratio for non-TMT zone	–0.083	0.394^e^	107
Mean ethanol spiking ratio for ethanol arms	–0.001	0.997^e^	31
Mean ethanol spiking ratio for non-ethanol zone	–0.041	0.822^e^	32
Peak ethanol spiking ratio for ethanol arms	–0.092	0.621^e^	31
Peak ethanol spiking ratio for non-ethanol zone	–0.062	0.734^e^	32
Mean extrafield TMT spiking ratio for TMT arms	–0.465	<0.001^e^	54
Peak extrafield TMT spiking ratio for TMT arms	–0.453	<0.00 ^e^	54
Mean intrafield TMT spiking ratio for TMT arms	0.013	0.925^e^	52
Peak intrafield TMT spiking ratio for TMT arms	–0.055	0.698^e^	52
Mean intrafield TMT spiking ratio for non-TMT zone	–0.024	0.864^e^	55
Peak intrafield TMT spiking ratio for non-TMT zone	–0.119	0.387^e^	55
Mean extrafield TMT spiking ratio for non-TMT zone	0.027	0.852^e^	52
Peak extrafield TMT spiking ratio for non-TMT zone	0.062	0.661^e^	52
Mean extrafield ChR2 spiking ratio for ChR2 arms	–0.429	0.007^e^	38
Peak extrafield ChR2 spiking ratio for ChR2 arms	–0.426	0.009^e^	38
Mean intrafield ChR2 spiking ratio for ChR2 arms	–0.261	0.099^e^	41
Peak intrafield ChR2 spiking ratio for ChR2 arms	–0.187	0.241^e^	41
Mean intrafield ChR2 spiking ratio for non-ChR2 zone	0.092	0.530^e^	49
Peak intrafield ChR2 spiking ratio for non-ChR2 zone	0.066	0.651^e^	49
Mean extrafield ChR2 spiking ratio for non-ChR2 zone	0.099	0.529^e^	42
Peak extrafield ChR2 spiking ratio for non-ChR2 zone	0.032	0.842^e^	42

Each spiking ratio is described with Pearson’s correlation (*r*), statistical significance (*p* value), and sample size (*n*).

### Extrafield spiking during aversion episodes determined the degree of field plasticity

**Figure 4.**
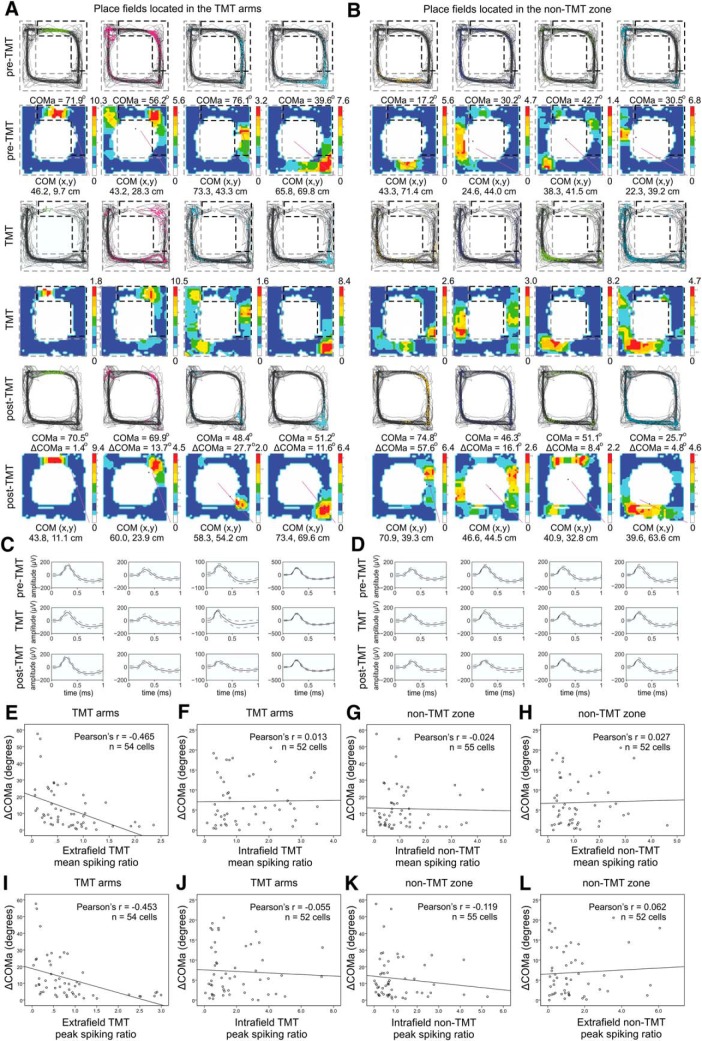
Increased extrafield place cell spiking during TMT exposure predicts spatial field reconfiguration. ***A***, Four place fields from sample rats with fields located in the TMT arms during pre-TMT sessions (top), TMT sessions (middle), and post-TMT sessions (bottom). For each session, the upper panels show the animal trajectory with spikes, marked with colored dots, while the lower panels show color-coded firing rate. ***B***, Four place fields from the same animal with fields outside the TMT arms. ***C***, Waveforms of the place cells from the pre-TMT, TMT, and post-TMT sessions shown in ***A***. ***D***, Waveforms of the cells shown in ***B***. The solid line shows the average waveform shape; the dashed lines show the 1 SD confidence intervals. ***E***, Correlation between ΔCOMa and mean TMT spiking ratio for the place cells located outside the TMT arms (extrafield spikes; Extended Data Fig. 4-1). ***F***, Correlation between ΔCOMa and mean TMT spiking ratio for the place cells located inside the TMT arms (intrafield spikes; Extended Data Fig. 4-2). ***G***, Correlation between ΔCOMa and mean non-TMT spiking ratio for the place cells located inside the non-TMT arms (intrafield spikes; Extended Data Fig. 4-3). ***H***, Correlation between ΔCOMa and mean non-TMT spiking ratio for the place cells located outside the non-TMT zone (extrafield spikes; Extended Data Fig. 4-4). ***I***, As for ***E***, but with peak TMT spiking ratio instead of mean TMT spiking ratio (Extended Data Fig. 4-1). ***J***, As for ***F***, but with peak TMT spiking ratio instead of mean TMT spiking ratio (Extended Data Fig. 4-2). ***K***, As for ***G***, but with peak non-TMT spiking ratio instead of mean non-TMT spiking ratio (Extended Data Fig. 4-3). ***L***, As for ***H***, but with peak non-TMT spiking ratio instead of mean non-TMT spiking ratio (Extended Data Fig. 4-4).

We next asked whether the spiking of the place cells with fields located in the TMT arms affects differently the TMT-induced field remapping compared to the spiking of the cells with fields located outside the TMT arms. We compared the remapping of place cells with fields located in the TMT arms (with spiking ratio including intrafield spikes; Fig. 4*A*) to the remapping of the cells with place fields outside the TMT arms (with spiking ratio including only extrafield spikes; Fig. 4*B*). The place field was defined as the region of the track in which the firing rate of the place cell was > 20% of the peak firing frequency (Brun et al., 2002). Mean and peak extrafield TMT spiking ratios were both strongly correlated with ΔCOMa [*r* = –0.465, *p* < 0.001^e^, *n* = 54 for mean TMT firing ratio (Fig. 4*E*, Extended Data Fig. 4-1); *r* = –0.453, *p* < 0.001^e^, *n* = 54 for peak TMT firing ratio (Fig. 4*I*)]. However, for intrafield TMT spiking ratios, there were no significant correlations with ΔCOMa for either the mean (Fig. 4*F*; Table 3, Extended Data Fig. 4-2) or peak ratios (Fig. 4*J*; Table 3). The dissociation of the spikes into extra- and intrafield had no effect on ΔCOMa analysis outside the TMT arms. In the non-TMT zone no significant correlation was evident for the mean and peak firing rate of the intra- (Fig. 4*G*,*K*; Table 3, Extended Data Fig. 4-3) and extrafield spikes (Fig. 4*H*,*L*; Table 3, Extended Data Fig. 4-4). These results show that the degree of increase in extrafield spiking during the TMT session predicted the degree of place field plasticity, whereby a higher increase predicted a greater plasticity field remapping.

10.1523/ENEURO.0423-17.2019.f4-1 Extended Data Figure 4-1 Extrafield TMT spiking ratio and ΔCOMa of the place cells’ spikes in TMT arms. Download Figure 4-1, DOCX file.

10.1523/ENEURO.0423-17.2019.f4-2 Extended Data Figure 4-2 Intrafield TMT spiking ratio and ΔCOMa of the place cells’ spikes in TMT arms. Download Figure 4-2, DOCX file.

10.1523/ENEURO.0423-17.2019.f4-3 Extended Data Figure 4-3 Intrafield TMT spiking ratio and ΔCOMa of the place cells’ spikes in the non-TMT zone. Download Figure 4-3, DOCX file.

10.1523/ENEURO.0423-17.2019.f4-4 Extended Data Figure 4-4 Extrafield TMT spiking ratio and ΔCOMa of the place cells’ spikes in the non-TMT zone. Download Figure 4-4, DOCX file.

### The COM shift occurs in both directions of navigation along the track

**Figure 5.**
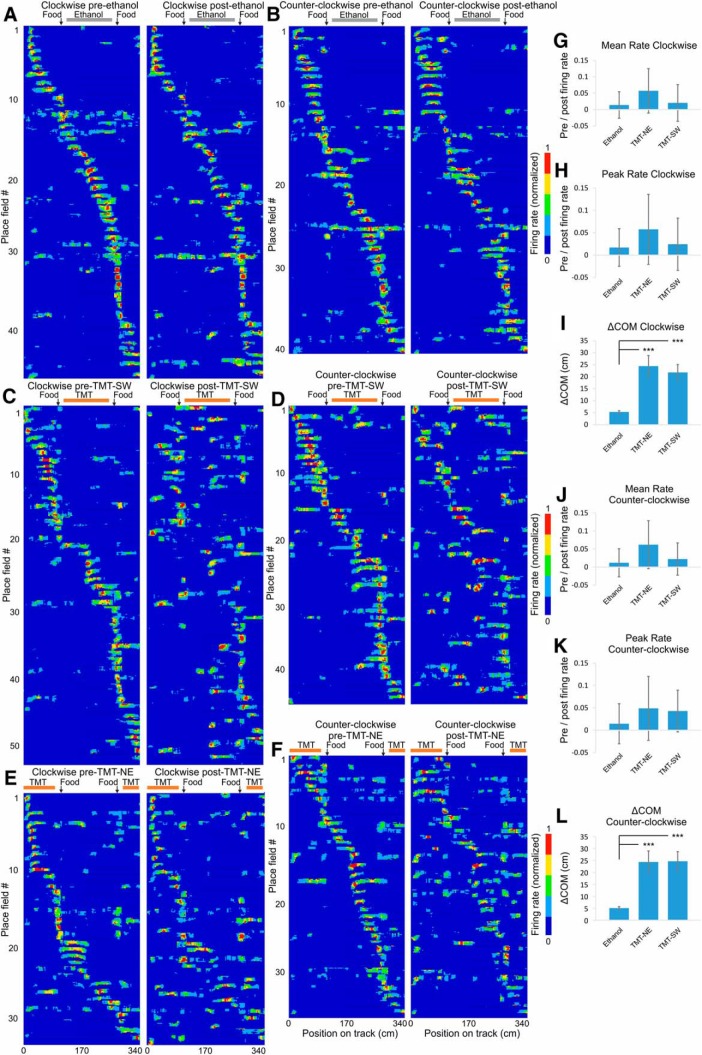
Field but not rate remapping after aversive experience. ***A***, ***B***, Color-coded linearized map showing the location of CA1 place fields before (left panel) and after (right panel) exposure to ethanol for clockwise (***A***; Extended Data Fig. 5-1) and counter-clockwise direction of movement (***B***; Extended Data Fig. 5-2). Each line shows the activity of one place cell (86 datasets in total from 63 place cells). The horizontal gray bar indicates the ethanol zone during the exposure session, while black vertical arrows indicate the location of the food delivery. ***C***, ***D***, Linearized map before (left panel) and after (right panel) exposure to TMT in the SW section of the track for clockwise (***C***; Extended Data Fig. 5-3) and counter-clockwise direction of movement (***D***; Extended Data Fig. 5-4). Each line shows the activity of one place cell (98 datasets in total from 66 place cells). The horizontal red bar indicates the TMT zone during the exposure session, while black vertical arrows indicate the location of the food delivery. ***E***, ***F***, Linearized maps before (left panel) and after (right panel) exposure to TMT in the NE section of the track for clockwise (***E***; Extended Data Fig. 5-5) and counter-clockwise direction of movement (***F***; Extended Data Fig. 5-6). Each line shows the activity of one place cell (68 datasets in total from 40 place cells). ***G*,** Comparison of the place field mean spiking before and after exposure to TMT. The pre/post normalized count represents a decrease (positive) and increase (negative values) for the mean firing rate of clockwise fields for ethanol, TMT-NE, and TMT-SW groups. Error bars, mean ± SEM. ***H*,** Comparison of the place field peak spiking before and after exposure to TMT of clockwise fields for the same groups. Error bars, mean ± SEM. ***I*,** COM shift (ΔCOM) after exposure to TMT of clockwise fields for ethanol, TMT-NE, and TMT-SW groups. Error bars, mean ± SEM; ***p < 0.001. ***J*,** Comparison of the place field mean spiking before and after exposure to TMT for counter-clockwise fields. Error bars, mean ± SEM. ***K*,** Comparison of the place field peak spiking before and after exposure to TMT for counter-clockwise fields. Error bars, mean ± SEM. ***L*,** ΔCOM after exposure to TMT for counter-clockwise fields. Error bars, mean ± SEM; ***p < 0.001.

We next aimed to precisely identify the degree of place field COM shift between recording sessions. The angle calculation of COM in 2D space is prone to higher variability compared to the calculation of COM in one dimension, because the distance covered per unit angle varies as a function of the radial distance. We therefore calculated field and rate remapping after the path of the animals was linearized (Fig. 5). Place fields on linear tracks may differ depending on the direction of the animal’s movement (McNaughton et al., 1983). Thus, we also separated the place fields into clockwise and counter-clockwise trajectories (Fig. 5*A*,*C*,*E* and *B*,*D*,*F*). We defined these unidirectional place fields using a different minimum firing rate cut-off to the one used in previous experiments, specifically, 10% of the maximum firing rate; 10% cut-off preserves more the peripheral spiking activity buts also increases the extrafield noise compared to 20% cut-off approach. We compared the spiking parameters of the place fields between: (1) pre-ethanol session (Fig. 5*A*,*B*, left panels) and post-ethanol session (Fig. 5*A*,*B*, right panels, Extended Data Figs 5-1 and 5-2); (2) pre-TMT session (Fig. 5*C*,*D*, left panels) and post-TMT session (Fig. 5*C*,*D*, right panels, Extended Data Figs 5-3 and 5-4) for the SW group; and (3) pre-TMT session (Fig. 5*E*,*F*, left panels) and post-TMT session (Fig. 5*E*,*F*, right panels, Extended Data Figs 5-5 and 5-6) for the NE group. There was no significant difference in the change in the mean firing rate among the ethanol, TMT-NE, and TMT-SW groups, either for the clockwise (ANOVA, *n* = 6, *F*_(2,128)_ = 0.257, *p* = 0.773^f^; Fig. 5*G*) or counter-clockwise fields (ANOVA, *n* = 6, *F*_(2,118)_ = 0.164, *p* = 0.849^f^; Fig. 5*J*). Similarly, there was no significant change in the peak firing rate between the three groups for the clockwise (ANOVA, *n* = 6, *F*_(2,128)_ = 0.886, *p* = 0.886^f^; Fig. 5*H*) or the counter-clockwise fields (ANOVA, *n* = 6, *F*_(2,118)_ = 0.118, *p* = 0.889^f^; Fig. 5*K*). The linearized COM shifted on average to 5.10 ± 0.5 cm for the clockwise fields (Fig. 5*I*) and 5.37 ± 0.5 cm for the counter-clockwise fields (Fig. 5*L*) in the ethanol-exposed group. Exposure to TMT evoked ΔCOM values of 24.24 ± 4.6 cm for clockwise fields and 24.47 ± 4.4 cm for counter-clockwise fields for the TMT-NE group and 21.84 ± 3.3 cm for clockwise fields (Fig. 5*I*) and 24.65 ± 4.0 cm for counter-clockwise fields (Fig. 5*L*) for the TMT-SW group. This shift was significantly higher than that of the control group for clockwise (ANOVA, *n* = 6, *F*_(2,128)_ = 11.52, *p* < 0.001^f^) and counter-clockwise fields (ANOVA, *n* = 6, *F*_(2,118)_ = 10.105, *p* < 0.001^f^). These results show that TMT evoked potent field remapping and negligible rate remapping for both directions of navigation.

10.1523/ENEURO.0423-17.2019.f5-1 Extended Data Figure 5-1 Unidirectional ethanol spiking comparison and ΔCOM for clockwise fields. Download Figure 5-1, DOCX file.

10.1523/ENEURO.0423-17.2019.f5-2 Extended Data Figure 5-2 Unidirectional ethanol spiking comparison and ΔCOM for counter-clockwise fields. Download Figure 5-2, DOCX file.

10.1523/ENEURO.0423-17.2019.f5-3 Extended Data Figure 5-3 Unidirectional TMT-SW spiking comparison and ΔCOM for clockwise fields. Download Figure 5-3, DOCX file.

10.1523/ENEURO.0423-17.2019.f5-4 Extended Data Figure 5-4 Unidirectional TMT-SW spiking comparison and ΔCOM for counter-clockwise fields. Download Figure 5-4, DOCX file.

10.1523/ENEURO.0423-17.2019.f5-5 Extended Data Figure 5-5 Unidirectional TMT-NE spiking comparison and ΔCOM for clockwise fields. Download Figure 5-5, DOCX file.

**Figure 6.**
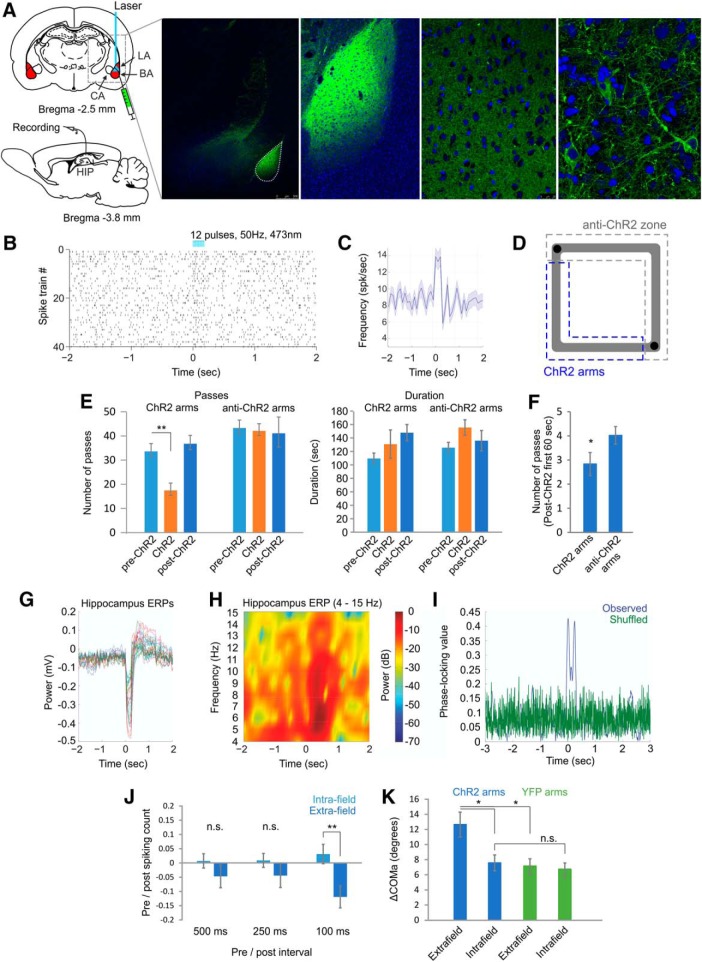
Photostimulation of the basolateral complex of the amygdala evokes spatial aversion. ***A***, Atlas schematic shows the injection site of AAV-CaMKIIα-hChR2-YFP and the optic fiber location in the basolateral complex of amygdala (BA, basal nucleus; LA, lateral nucleus of amygdala; CA, central nucleus of amygdala). The confocal images on the right show increasing levels of magnification of the YFP-expressing neurons (green) in the BLA is histology with DAPI staining (blue). ***B***, ***C***, Raster plot from 40 repetitions (***B***) and averaged firing frequency (***C***) of optically evoked time-locked excitation of a BLA cell. Time 0 indicates the delivery of the first train of the stimulation protocol. ***D***, Experimental setup: light delivery in the SW ChR2 arms, marked with dashed blue line. ***E***, Number of passes (left panel) and duration in seconds (right panel) counted in the ChR2 arms versus non-ChR2 arms during the pre-ChR2, ChR2, and post-ChR2 sessions; **p < 0.01. Error bars, mean ± SEM. ***F***, Number of passes through the ChR2 and non-ChR2 arms for the first 60 s of the post-ChR2 session. ***G***, Event-related potentials (ERPs) recorded in dorsal CA1 from 32 electrodes in a sample animal. Time 0 indicates the delivery of the onset of BLA optogenetic stimulation. ***H***, Color-coded power spectrogram of hippocampal low-frequency oscillations (4–15 Hz) during the photostimulation protocol. ***I***, Representative phase-locking value after BLA photostimulation for the observed data (blue) and for control shuffled data (green). ***J***, Comparison of the place cell’s spiking before and after the photostimulation onset for intrafield and extrafield spikes. The pre/post normalized count represents a decrease (positive) and increase (negative values) for the first 500 ms (left), 250 ms (middle), and 100 ms (right) after the optogenetic protocol onset for the intrafield (light blue) and extrafield (blue) spikes; **p < 0.01. Error bars, mean ± SEM. ***K***, ΔCOMa for place fields located outside the ChR2 arms (extrafield) and for place cells located inside the ChR2 arms (intrafield) from ChR2 recordings (blue) and control YFP recordings (green); *p < 0.05. Error bars, mean ± SEM.

10.1523/ENEURO.0423-17.2019.f5-6 Extended Data Figure 5-6 Unidirectional TMT-NE spiking comparison and ΔCOM for counter-clockwise fields. Download Figure 5-6, DOCX file.

### BLA photostimulation mediated an aversion-triggered shift in the COM

BLA activation is known to trigger aversive behavior (Davis, 1992). We investigated whether activation of BLA evokes aversion-induced field plasticity of hippocampal place cells and whether optogenetic activation of BLA excitatory neurons would exert an effect similar to the TMT-evoked pattern of reconfiguration. To exert spatial control of BLA neuronal activity, we injected the BLA (Fig. 6*A*) with the viral construct AAV-CaMKIIα-hChR2-YFP, which expresses ChR2 in BLA pyramidal neurons driven by α-calcium/calmodulin-dependent kinase II promoter (Bukalo et al., 2015). Delivery of blue light (473 nm) elicited the spiking of neurons infected with AAV-CaMKIIα-hChR2-YFP (Fig. 6*B*,*C*) and concurrently induced aversion behavior (Movies 2, 3). The majority of the photostimulated BLA neurons were CamKIIα-positive; 87 ± 8% of neurons that expressed YFP also expressed CamKIIα, while 64 ± 5% of neurons that expressed CamKIIα also expressed YFP. We applied optogenetic stimulation (50 Hz, trains of 12 pulses, 0.5-Hz intertrain interval, 473 nm) in the SW arms of the track (ChR2 arms; Fig. 6*D*), and observed place avoidance in these arms (*t* test, *n* = 6 rats, *t*_(5)_ = 5.0, *p* = 0.004^a^; Fig. 6*E*; Table 2). Place aversion was similarly observed in the first minute of the post-ChR2 session (*t* test, *n* = 6, *t*_(5)_ = –3.796, *p* = 0.013^a^; Fig. 6*F*). BLA photostimulation augmented the synchronization of hippocampal local field oscillations across the stimulation trials (Fig. 6*G*), where the power of the event-related potential increased for the frequency range of 5–8 Hz (Fig. 6*H*). The phase-locking value quantifies the degree of local field synchrony among all stimulation epochs (see Materials and Methods). Phase-locking values were significantly higher for BLA light pulse delivery (0.35 ± 0.04; Fig. 6*I*) compared to both the shuffled BLA data (0.09 ± 0.07, *t* test, *n* = 120 trials, *t*_(119)_ = 3.0, *p* = 0.005^a^) and the control YFP group, injected with virus bearing only the YFP reporter (0.01 ± 0.06, *t* test, *n* = 120 trials, *t*_(118)_ = 4.8, *p* = 0.009^d^). These results show that although there is no direct projection from BLA to dorsal hippocampus, the activation of BLA neurons indirectly evokes potent network response in the CA1 region of dorsal hippocampus.

Movie 2. **ChR2-induced aversion.** Navigation on a rectangular-shaped linear track by a rat with chronically implanted tetrodes in the hippocampus. The first half of the video shows a pre-ChR2 session, measuring baseline behavioral and electrophysiological activity signals. The second half of the video shows light delivery, triggering photoexcitation of BLA neurons. The coordinates of photostimulation (ChR2 arms) are denoted in blue. 10.1523/ENEURO.0423-17.2019.movie.2

Movie 3. **ChR2-induced aversion.** Pre-ChR2 and ChR2 sessions for another animal. 10.1523/ENEURO.0423-17.2019.movie.3

Earlier data show that the magnitude of place field remapping after exposure to TMT depends on the degree of the extrafield but not intrafield spiking. Thus, we next examined whether BLA photostmulation affects differently extrafield and intrafield spiking. We compared the changes in the intrafield and extrafield spiking of place cells during the photostimulation of BLA. The spike count was examined in epochs of 100-, 250-, and 500-ms pre-BLA and post-BLA stimulation onset. The positive value of the normalized spike count (0.03 ± 0.03) shows that the activation of BLA resulted in a tendency for a decreased intrafield spiking rate in the first 100-ms post-BLA photostimulation (Fig. 6*J*). Concurrently, the extrafield spiking increased (represented by the negative value of the normalized spike count, –0.11 ± 0.04), which was significantly different from the extrafield normalized spike count for the first 100-ms (*t* test, *n* = 74 cells, *t*_(73)_ = 3.2, *p* = 0.002^a^) but not the 250-ms (*t* test, *n* = 74 cells, *t*_(73)_ = 1.1, *p* = 0.254^a^) or 500-ms post-BLA (*t* test, *n* = 74 cells, *t*_(73)_ = 1.2, *p* = 0.247^a^) photostimulation. This finding shows that amygdalar activity exerts differential effect on hippocampal place cells’ spiking, whereby intrafield spiking decreases and extrafield spiking increases in the first 100 ms after BLA activation.

**Figure 7.**
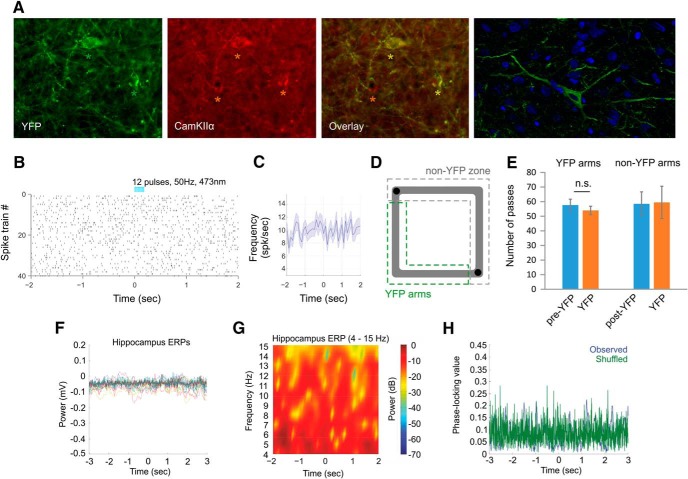
Photostimulation of control YFP-expressing BLA neurons. ***A***, YFP expression (left), anti-calcium/calmodulin-dependent protein kinase IIα (anti-CamKIIα) staining (middle) and their overlay in the BLA (right). The green asterisks show two YFP-expressing neurons, and the red asterisks show three CamKIIα-marked neurons. The confocal image on the far right shows YFP-expressing neurons (green) in the BLA with DAPI staining (blue). Raster plot from 40 repetitions (***B***) and firing frequency (***C***) of time-locked photostimulation of a BLA cell in control animals injected with AAV-YFP viral construct. Time 0 indicates the delivery of the first train of the stimulation protocol. ***D***, Experimental setup: light delivery in the SW arms of the rectangular-shaped linear track (YFP arms, marked with dashed green line). No photostimulation was applied in the non-ChR2 zone (marked with dashed gray line). ***E***, Number of passes of control animals injected with AVV-YFP construct in the YFP arms versus non-YFP zone during the pre-YFP, YFP, and post-YFP sessions. Error bars, mean ± SEM. ***F***, Event-related potentials (ERPs) recorded in dorsal CA1 from 32 electrodes in a sample control animal. Time 0 indicates the delivery of the onset of BLA YFP photostimulation. ***G***, Color-coded power spectrogram of hippocampal low-frequency oscillations (4–15 Hz) during the YFP photostimulation. ***H***, Representative phase-locking value after BLA YFP photostimulation for the observed data (blue) and for control shuffled data (green).

**Figure 8.**
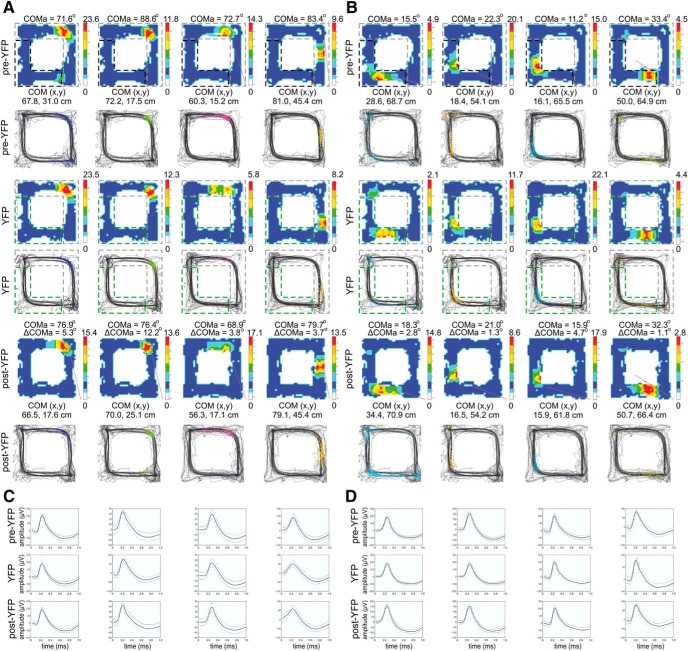
Control place fields during BLA photostimulation. ***A***, Four place fields from two sample animals with fields located outside the YFP arms during pre-YFP sessions (top), YFP sessions (middle), and post-YFP sessions (bottom). For each session, the upper panels show the color-coded firing rate, while the lower panels show the animal trajectory with spikes, marked with colored dots. ***B***, Four place fields from the same two animals with fields located inside the YFP arms during pre-YFP sessions (top), YFP sessions (middle), and post-YFP sessions (bottom). ***C***, Waveforms of the place cells shown in ***A***. ***D***, Waveforms of the cells shown in ***B***. The solid line shows the average waveform shape; the dashed lines show the 1 SD confidence intervals.

As described earlier for TMT, we then compared ΔCOMa between the pre-ChR2 and post-ChR2 sessions for the place cells with fields located in the ChR2 arms (with intrafield spikes) and for the place cells with fields located in the non-ChR2 zone (with extrafield spikes only). The average ΔCOMa of 7.57 ± 1.1° from cells with fields located in the ChR2 arms was significantly lower than ΔCOMa of 12.64 ± 1.6° from cells located in the non-ChR2 zone (*t* test, extrafield group, *n* = 38 cells, intrafield group, *n* = 42 cells, *t*_(78)_ = 5.0, *p* = 0.011^d^; Fig. 6*K*). ΔCOMa for the extrafield spiking cells also differed significantly between the ChR2- and control YFP groups of rats (injected with AAV-CaMKIIα-YFP) where ΔCOMa for the YFP group was 6.66 ± 0.9° (*t* test, ChR2 extrafield group, *n* = 38 cells, YFP extrafield group, *n* = 32 cells, *t*_(68)_ = 5.3, *p* = 0.010^d^). BLA photostimulation of the control YFP group of rats (Fig. 7*A*) evoked no behavioral response (*t* test, *n* = 6 rats, *t*_(5)_ = 1.0, *p* = 0.358^a^; Fig. 7*D*,*E*), electrophysiological response (Fig. 7*B*,*C*,*F–H*) or remapping (Fig. 8*A*,*B*). These results show that depolarization of BLA neurons triggers larger degree of place field remapping when paired with intrafield spiking compared to BLA depolarization paired with extrafield spiking.

### Extrafield spiking during BLA photostimulation predicts the COM shift

**Figure 9.**
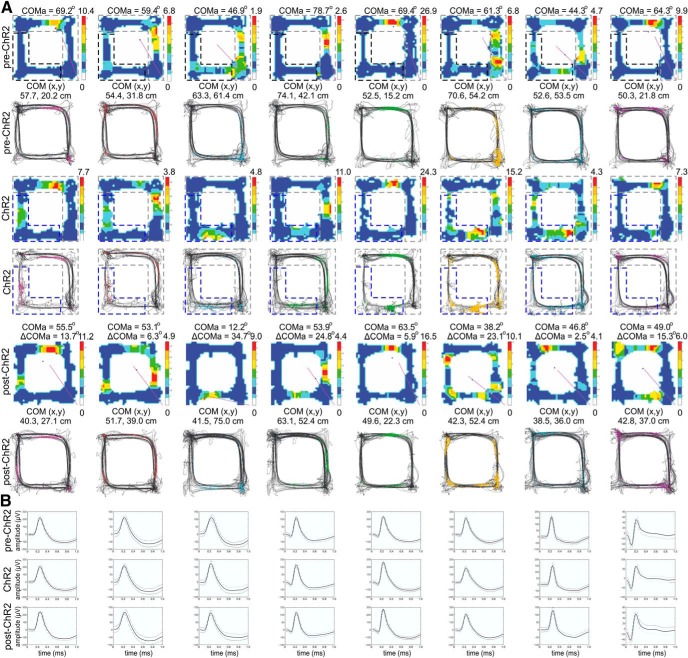
Place fields of cells with high extrafield spiking during BLA photostimulation. ***A***, Eight place cells from four rats, with fields located outside the ChR2 arms, with expressed extrafield spiking activity during the light delivery. Top panels, Pre-ChR2 session. Middle panels, ChR2 session. Bottom panels, Post-ChR2 session. For each session, the upper panels show the color-coded firing rate, while the lower panels show the animal trajectory with spikes, marked with colored dots. ***B***, Waveforms of the place cells above, recorded from the pre-ChR2 (top row), ChR2 (middle row), and post-ChR2 (bottom row) session. The solid line shows the average waveform shape; the dashed lines show the 1 SD confidence intervals.

**Figure 10.**
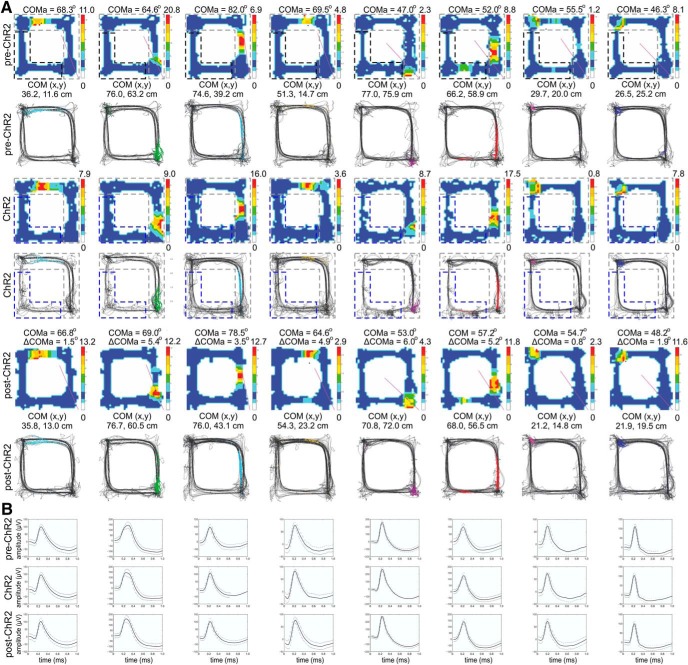
Place fields of cells with low extrafield spiking during BLA photostimulation. ***A***, Eight place cells from the same four rats, with fields located outside the ChR2 arms, with little or no extrafield spiking activity during the light delivery. For each session, the upper panels show the color-coded firing rate, while the lower panels show the animal trajectory with spikes, marked with colored dots. ***B***, Waveforms of the place cells above, recorded from the pre-ChR2 (top row), ChR2 (middle row), and post-ChR2 (bottom row) session. The solid line shows the average waveform shape; the dashed lines show the 1 SD confidence intervals.

**Figure 11.**
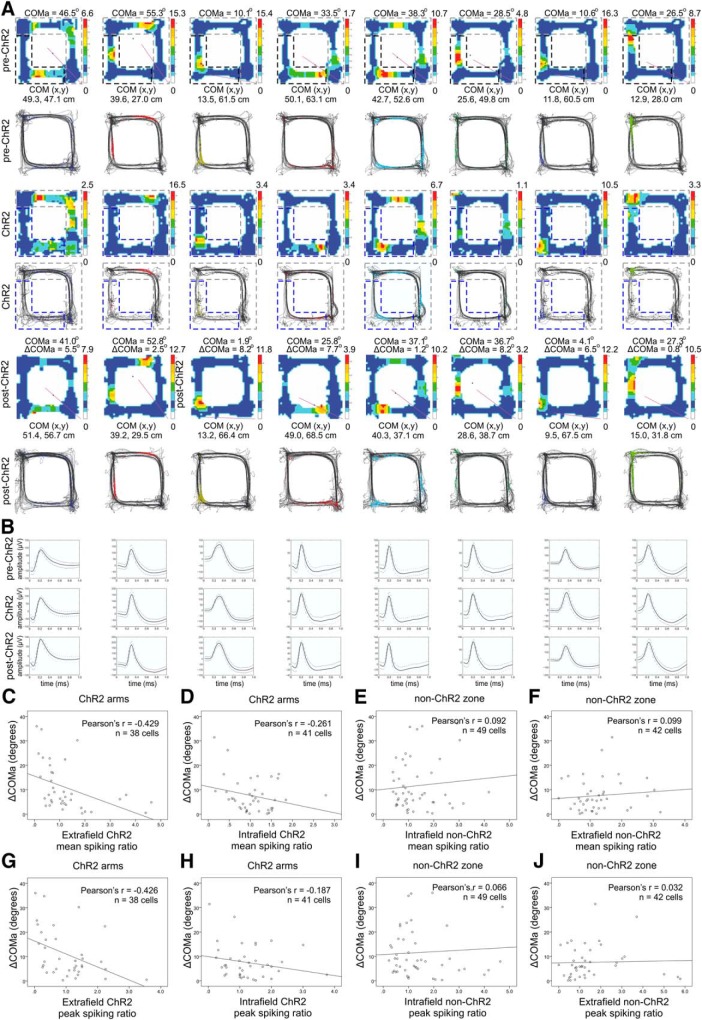
The BLA-triggered hippocampal spiking increase correlates with the place field COM shift. ***A***, Eight place cells from four rats, with fields located inside the ChR2 arms, with intrafield spiking during the light delivery. Top panels, Pre-ChR2 session. Middle panels, ChR2 session. Bottom panels, Post-ChR2 session. For each session, the upper panels show the color-coded firing rate, while the lower panels show the animal trajectory with spikes, marked with colored dots. ***B***, Waveforms of the place cells above, recorded from the pre-ChR2 (top row), ChR2 (middle row), and post-ChR2 (bottom row) session. The solid line shows the average waveform shape; the dashed lines show the 1 SD confidence intervals. ***C***, Correlation between ΔCOMa and mean ChR2 spiking ratio for the extrafield spikes of the place cells located outside the ChR2 arms (Extended Data Fig. 11-1). ***D***, Correlation between ΔCOMa and mean ChR2 spiking ratio for the intrafield spikes of the place cells located inside the ChR2 arms (Extended Data Fig. 11-2). ***E***, Correlation between ΔCOMa and mean non-ChR2 spiking ratio for the intrafield spikes of the place cells located inside the non-ChR2 zone (Extended Data Fig. 11-3). ***F***, Correlation between ΔCOMa and mean non-ChR2 spiking ratio for the extrafield spikes of the place cells located outside the non-ChR2 zone (Extended Data Fig. 11-4). ***G***, As for ***C***, but with peak ChR2 spiking ratio instead of mean ChR2 spiking ratio (Extended Data Fig. 11-1). ***H***, As for ***D***, but with peak ChR2 spiking ratio instead of mean ChR2 spiking ratio (Extended Data Fig. 11-2). ***I***, As for ***E***, but with peak non-ChR2 spiking ratio instead of mean ChR2 spiking ratio (Extended Data Fig. 11-3). ***J***, As for ***F***, but with peak non-ChR2 spiking ratio instead of mean ChR2 spiking ratio (Extended Data Fig. 11-4).

Our next goal was to establish whether the BLA photostimulation shows similar to the TMT-induced pattern of remapping. Thus, we examined if the degree of BLA-triggered place field plasticity correlates to an increase in extrafield but not intrafield spiking activity. Place fields of the cells located in the non-ChR2 zone with extrafield spikes during the photostimulation session (Fig. 9) showed large shifts in their COM (Movie 4), while the cells with little or no extrafield spiking activity showed small changes in their COM (Fig. 10). Concurrently, minor changes in the COM were evident for the place cells located in the ChR2 arms (with intrafield spikes; Fig. 11*A*). We obtained correlations between ΔCOMa and the ratio of the baseline over the ChR2 session place cells’ firing rate from the recorded spikes (ChR2 spiking ratio). The mean and the peak extrafield ChR2 spiking ratios for the ChR2 arms were significantly correlated with ΔCOMa [*r* = –0.429, *p* = 0.007^e^, *n* = 38 (Fig. 11*C*, Extended Data Fig. 11-1); *r* = –0.426, *p* = 0.009^e^, *n* = 38 (Fig. 11*G*)]. The mean and the peak intrafield ChR2 spiking ratio showed a weak nonsignificant correlation with ΔCOMa for the ChR2 arms (Fig. 11*D*,*H*; Table 3, Extended Data Fig. 11-2). There was no significant correlation between ΔCOMa and the mean and peak firing rate of the intra- (Fig. 11*E*,*I*; Table 3, Extended Data Fig. 11-3) and extrafield spikes (Fig. 11*F*,*J*; Table 3, Extended Data Fig. 11-4) for the non-ChR2 zone. Similarly to the TMT session results, BLA photostimulation resulted in a significant negative correlation between the spiking ratio and ΔCOMa for the ChR2 arms only, and only for the extrafield spiking cells. These results support the hypothesis that aversion-evoked place field reconfiguration is mediated by BLA activation.

10.1523/ENEURO.0423-17.2019.f11-1 Extended Data Figure 11-1 Extrafield ChR2 spiking ratio and ΔCOMa of the place cells’ spikes in ChR2 arms. Download Figure 11-1, DOCX file.

10.1523/ENEURO.0423-17.2019.f11-2 Extended Data Figure 11-2 Intrafield ChR2 spiking ratio and ΔCOMa of the place cells’ spikes in ChR2 arms. Download Figure 11-2, DOCX file.

10.1523/ENEURO.0423-17.2019.f11-3 Extended Data Figure 11-3 Intrafield ChR2 spiking ratio and ΔCOMa of the place cells’ spikes in the non-ChR2 zone. Download Figure 11-3, DOCX file.

10.1523/ENEURO.0423-17.2019.f11-4 Extended Data Figure 11-4 Extrafield ChR2 spiking ratio and ΔCOMa of the place cells’ spikes in the non-ChR2 zone. Download Figure 11-4, DOCX file.

Movie 4. **Extrafield spiking during ChR2 photostimulation of BLA.** Analysis software visualization of the animal’s path (marked with black line) and the recorded place cell’s spiking (denoted with purple dots). The represented speed is 2× real time. The first half of the video shows 238 s of a pre-ChR2 session, followed by images of the raw signal and the firing map for the entire 12-min baseline session. The second half of the video shows 238 s of a ChR2 session, followed by images of the raw signal and the firing map for the entire 12-min ChR2 session. The coordinates of photostimulation (ChR2 arms) are denoted in blue. 10.1523/ENEURO.0423-17.2019.movie.4

### The unidirectional field shift after BLA photostimulation is similar to TMT-induced remapping

**Figure 12.**
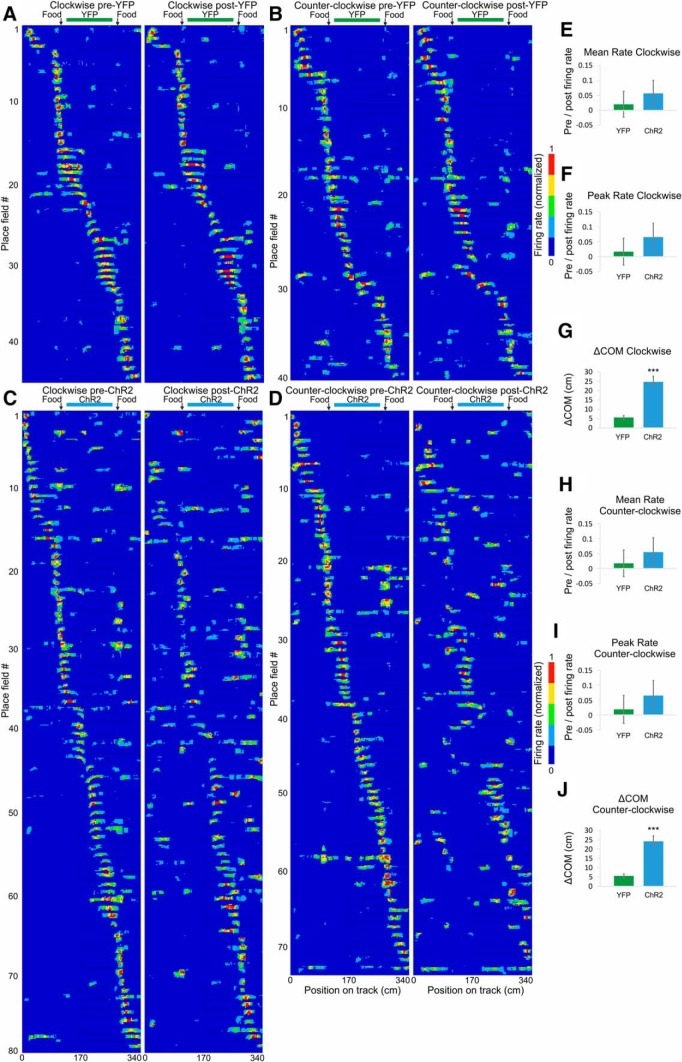
Unidirectional field remapping after BLA photostimulation. ***A***, ***B***, Color-coded linearized map showing location of CA1 place fields before (left panel) and after (right panel) light delivery session to the YFP group of rats for clockwise (***A***; Extended Data Fig. 12-1) and counter-clockwise direction of movement (***B***; Extended Data Fig. 12-2). Each line shows the activity of one place cell (84 datasets in total from 69 place cells). The horizontal green bar indicates the light delivery YFP zone during the exposure session, and black vertical arrows indicate the location of the food delivery. ***C***, ***D***, Color-coded linearized map showing the location of CA1 place fields before (left panel) and after (right panel) light delivery session to the ChR2 group of rats for clockwise (***C***; Extended Data Fig. 12-3) and counter-clockwise direction of movement (***D***; Extended Data Fig. 12-4). Each line shows the activity of one place cell (154 datasets in total from 79 place cells). The horizontal blue bar indicates the light delivery ChR2 zone during the exposure session, while black vertical arrows indicate the location of the food delivery. ***E*,** Comparison of the place field mean spiking before and after BLA photostimulation. The pre/post normalized count represents a decrease (positive) and increase (negative values) for the mean firing rate of clockwise fields for the YFP and ChR2 groups. Error bars, mean ± SEM. ***F*,** Comparison of the place field peak spiking before and after BLA photostimulation of clockwise fields for the same groups. Error bars, mean ± SEM. ***G***, COM shift (ΔCOM) after BLA photostimulation of clockwise fields for YFP and ChR2 groups. Error bars, mean ± SEM; ***p < 0.001. ***H*,** Comparison of the place field mean spiking before and after BLA photostimulation for counter-clockwise fields. Error bars, mean ± SEM. ***I*,** Comparison of the place field peak spiking before and after BLA photostimulation for counter-clockwise fields. Error bars, mean ± SEM. ***J*,** COM shift (ΔCOM) after BLA photostimulation for counter-clockwise fields. Error bars, mean ± SEM; ***p < 0.001.

Next, we examined the spiking parameters of the linearized place fields of the ChR2 group of rats and YFP control animals. We compared the change in COM and firing rate between the pre-YFP session (Fig. 12*A*,*B*, left panels) and the post-YFP session (Fig. 12*A*,*B*, right panels, Extended Data Figs 12-1 and 12-2), as well as pre-ChR2 session (Fig. 12*C*,*D*, left panels) and post-ChR2 session (Fig. 12*C*,*D*, right panels, Extended Data Figs 12-3 and 12-4). There was no significant difference in the change in the mean firing rate between the YFP and ChR2 groups for the clockwise (ANOVA, *n* = 6, *F*_(1,122)_ = 0.269, *p* = 0.847^f^; Fig. 12*E*) and counter-clockwise groups (ANOVA, *n* = 6, *F*_(1,112)_ = 0.278, *p* = 0.810^f^; Fig. 12*H*). No significance was evident for the change in the peak firing rate between the two groups for either the clockwise (ANOVA, *n* = 6, *F*_(1,122)_ = 0.266, *p* = 0.850^f^; Fig. 12*F*) or counter-clockwise fields (ANOVA, *n* = 6, *F*_(1,112)_ = 0.126, *p* = 0.816^f^; Fig. 12*I*). The linearized ΔCOM was 5.66 ± 1.1 cm for the clockwise fields (Fig. 12*G*) and 5.61 ± 1.1 cm for the counter-clockwise fields (Fig. 12*J*) in the YFP group, which was significantly lower compared to the BLA group with ΔCOM of 24.60 ± 4.6 cm for clockwise fields (ANOVA, *n* = 6, *F*_(1,122)_ = 12.01, *p* < 0.001^f^) and 24.10 ± 3.0 cm for counter-clockwise fields (ANOVA, *n* = 6, *F*_(1,112)_ = 11.07, *p* < 0.001^f^). These results show that, similarly to TMT, BLA excitation resulted in a strong field remapping, but insignificant rate remapping for both directions of navigation.

10.1523/ENEURO.0423-17.2019.f12-1 Extended Data Figure 12-1 Unidirectional YFP spiking comparison and ΔCOM for clockwise fields. Download Figure 12-1, DOCX file.

10.1523/ENEURO.0423-17.2019.f12-2 Extended Data Figure 12-2 Unidirectional YFP spiking comparison and ΔCOM for counter-clockwise fields. Download Figure 12-2, DOCX file.

10.1523/ENEURO.0423-17.2019.f12-3 Extended Data Figure 12-3 Unidirectional ChR2 spiking comparison and ΔCOM for clockwise fields. Download Figure 12-3, DOCX file.

10.1523/ENEURO.0423-17.2019.f12-4 Extended Data Figure 12-4 Unidirectional ChR2 spiking comparison and ΔCOM for counter-clockwise fields. Download Figure 12-4, DOCX file.

## Discussion

In this study, we report that the place fields located outside the area of aversion express higher propensity for COM shift compared to the fields located within the area of TMT-induced aversion. For the first time, we showed that the degree of COM shift is correlated with the firing rate of the extrafield spiking during aversion. Optogenetic stimulation of BLA paired with hippocampal extrafield spikes resulted in the same reconfiguration pattern as exposure to an innately aversive odor.

### β Amplitude increase reveals the spatial location of aversive odor perception

Recent data demonstrate that hippocampal spatial representation is not static but alters in response to nonspatial rewarding stimuli (Mamad et al., 2017). Hippocampal place cells also remap after exposure to stressful or fearful events (Moita et al., 2004; Wang et al., 2012, 2015; Kim et al., 2015). However, there is no clear explanation why some neurons alter their place fields to encode fearful experience, while others preserve stable fields. The application of innate aversive TMT allowed us to study the transformation of hippocampal population code from indifferent to negative context value for a single environment. The advantage of TMT is the absence of a learning curve required for associative fear conditioning (Kobayakawa et al., 2007) and the absence of visible or tactile features of the stressor that might affect the stability of the recorded place cells (Jeffery and O'Keefe, 1999). β Rhythm is a characteristic oscillation in the olfactory bulb during odorant perception (Lowry and Kay, 2007; Lepousez and Lledo, 2013). We measured β oscillations to identify in which arms of the track the animals perceive the aversive odorant. This approach allowed us to distinguish whether TMT odor was processed by the hippocampal formation of the animals only for particular arms or for the entire rectangular-shaped linear track. Entorhinal–hippocampal coupling was observed specifically in the 20- to 40-Hz frequency band as rats learned to use an odor cue to guide navigational behavior (Igarashi et al., 2014). The essence of β rhythm in the processing of aversive stimuli across the limbic circuitry has been demonstrated with the finding that powerful β activity predicted the behavioral expression of conditioned odor aversion (Chapuis et al., 2009). Similarly, we showed that β amplitude increased during the TMT sessions, particularly in the TMT arms but not in the rest of the track. We found that β rhythm varied as a function of whole-body speed, whereby a faster whole-body motion resulted in a lower β frequency and an increased β amplitude. The frequency dependence on the speed of locomotion is characteristic for not only the β frequency band, but also for slower and faster local field oscillations. For example, an increase in γ activity has been linked to faster running speed in rats, and this phenomenon was proposed to preserve the spatial specificity of place cells at different running speeds (Ahmed and Mehta, 2012). Further research is needed to clarify the source of the currents underpinning β activity. Particularly intriguing is the open question of how hippocampal pyramidal cells discharge in relation to β phase during the onset of aversive experience. We need to elucidate the temporal link between β and θ as well as β and γ activity during the perception of aversive stimuli.

### Aversive experience reconfigures hippocampal place fields

The reconfiguration of individual place fields is best evaluated by the COM difference (Knierim, 2002). We found that place fields from animals exposed to TMT displayed a larger COM change than those of control animals. Exposure to predator odor produces remapping only to some place fields, and one possible explanation for this is that the remapping cells are sensitive to nonspatial contextual cues such as olfactory information and/or emotional valence (Wang et al., 2012). Another possibility is that spatial or temporal proximity of the neuronal activity to the perception of the aversive episode determines which place fields reconfigure. To show that the spatial proximity to the olfactory stimulus was an essential component of the field plasticity, we assessed the correlation between the change in the COMa (ΔCOMa) and TMT spiking ratio within the TMT arms versus outside the TMT arms (non-TMT zone). Surprisingly, we found that the place fields inside the TMT arms showed no significant correlation between their intrafield spiking and COM shift, while the place fields outside the TMT arms demonstrated a high correlation between their extrafield spiking and the COM shift. No significant correlation was found between the intrafield or extrafield spiking in the non-TMT zone of the track and COM shift of the place fields. Although our knowledge about the contribution of extrafield spikes to spatial navigation is lacking, we know that their activity is crucially involved in hippocampus-dependent learning (Ferguson et al., 2011). The significance of the extrafield spikes is largely associated with the sharp-wave ripple replay of recently navigated locations and experiences (Wu et al., 2017), which are important in learning and memory consolidation during inactive behavioral state (Johnson et al., 2009). However, extrafield spikes from place cells have been shown to occur during active navigation (Johnson and Redish, 2007; Epsztein et al., 2011). Here, we present evidence that extrafield activity encodes salient stimuli and that it is potent enough to evoke place field plasticity related to experience-dependent learning.

### Rewarding stimuli in the context of aversive experience

Not only aversion, but also reward and novelty salient stimuli, can trigger biased field distribution (Fyhn et al., 2007). Our experimental design included two feeding locations. Therefore, these may have contributed to the observed remapping. The delivery of sugar pellets ensured continuous navigation for 12 min for each recording session. To reduce the effect of this reward on place field remapping, we habituated the rats to the feeding locations. The animals were consistently rewarded throughout the baseline (pre-)recordings, TMT/ethanol or YFP/ChR2 recordings and subsequent (post-)recordings for all groups of animals. Our unidirectional field analysis revealed that the aversion (TMT or ChR2) groups displayed a significantly greater ΔCOM than the control (ethanol or YFP) groups. The observed field plasticity was field remapping but not rate remapping. However, this result does not rule out the possibility that this plasticity results from a change in reward value due to TMT exposure, rather than the mere effect of conditioned aversion. A study in behaving rats showed that a decrease in lever pressing for food in the presence of a conditioned tone also led to changes in firing rate in areas associated with fear learning (Sotres-Bayon et al., 2012). Therefore, the perception of known reward value may change due to conflict between aversion and reward. We must acknowledge the likelihood that the COM shift observed in the non-TMT (non-ChR2) arms may be the result of conflict between the rewarding and aversive stimuli. Another factor that can affect place field variability is the change in whole-body speed. Although the post-TMT (ChR2) sessions were characterized by variable speed during the first 2 min, the animals’ navigation recovered for the remaining 10 min. This is a sufficient duration for the reliable formation of stable fields (Frank et al., 2004). Even so, we excluded spikes that occurred during epochs with running speeds below 5 cm/s (Alme et al., 2014).

### Amygdalar activation can shift the place field COM

The amygdala is a key structure in the processing of aversive experience (LeDoux, 2000), and optogenetic stimulation of BLA mediates associative fear learning (Johansen et al., 2010; Klavir et al., 2017). The amygdala sets the emotional valence of sensory stimuli (Phelps and LeDoux, 2005; Moriceau et al., 2006), and BLA circuitry is particularly involved in odor-evoked fear conditioning (Wallace and Rosen, 2001; Anderson et al., 2003; de Araujo et al., 2003). The amygdala is critical for stress-induced modulation of hippocampal synaptic plasticity and hippocampus-dependent learning (Kim et al., 2001; Vouimba and Richter-Levin, 2005). Aversion-triggered place cell remapping is blocked by amygdala inactivation (Donzis et al., 2013; Kim et al., 2015), while BLA stimulation decreases the stability of CA1 place fields (Kim et al., 2012). Despite recent advances in manipulating engrams (Cai et al., 2016; Rashid et al., 2016), there is no agreement as to whether place cells remap randomly or according to the location of the aversive stimulus. Here, we demonstrate that the remapping of the place fields depends on the location of the aversive stimulus, and that this process is mediated by activation of BLA and concurrent hippocampal extrafield spiking. We found that the BLA-triggered field plasticity pattern was comparable to the TMT-induced field reconfiguration; namely, the correlation between the ChR2 spiking ratio and ΔCOMa was only significant for place cells located outside the ChR2 arms. Furthermore, the increase in extrafield but not intrafield spiking during aversive episodes predicted the change in the preferred firing location. Such a response can emerge after a small, spatially uniform depolarization (Rickgauer et al., 2014) of the spatially untuned somatic membrane potential of an inactive place cell leads to the sudden and reversible emergence of a spatially tuned subthreshold response and novel place field formation (Lee et al., 2012; Bittner et al., 2015). This phenomenon is proposed as a key mechanism for the formation of hippocampal memory representations (Lee et al., 2012). Our data complement these findings by showing that BLA stimulation only increased the extrafield and not the intrafield spiking of the CA1 place cells. Taken together, this suggests that extrafield spikes are highly susceptible to hippocampal inputs and mediate spike-timed synaptic plasticity that results in relocation of the place fields’ COM.

### Together, the amygdala and hippocampus mediate fear memory acquisition

Early studies showed that the amygdala can regulate the induction of hippocampal synaptic plasticity. Long-term potentiation in the dentate gyrus can be attenuated by lesion of the BLA (Ikegaya et al., 1994) or by pharmacological inactivation of the BLA (Ikegaya et al., 1995). Priming the BLA inputs 30 s prior to performant path stimulation resulted in the facilitation of the excitatory postsynaptic potentials in the dorsal hippocampus (Akirav and Richter-Levin, 1999). Thus, the BLA modulates synaptic plasticity within the hippocampal formation, where the amygdala and the hippocampus act synergistically, to form long-term memories of emotional events. The dual activation of the amygdala and the hippocampus and the cross-talk between them has been proposed to provide contextual information to emotionally-based memories (Richter-Levin and Akirav, 2000). Our results suggest that this process can be detected in the spatial firing patterns of the place cells, which shift their COM towards the aversive section of the environment. While our experimental design involved brief aversion retrieval (during the first minute of the post-TMT/ChR2 sessions) followed by aversion extinction, the observed field reconfiguration may be specific for the extinction phase. The place field remapping pattern between the retrieval and extinction may differ (Wang et al., 2015). However, the long-term measurement of place cell activity during aversion retrieval is challenging due to the risk of navigation undersampling, which is a reason for incomplete formation of place fields per se and invalidates the evaluation of their properties (Hok et al., 2012; Navratilova et al., 2012).

### Contextual signaling across the hippocampal formation

Although the physiology of the dorsal hippocampus is regulated by aversive amygdalar signals, it is still unclear, which is the most direct anatomical route from the BLA to the dorsal hippocampus. The most substantial projection to the hippocampus originates in the basal nucleus and the caudomedial portion of the BLA projects heavily to the stratum oriens and stratum radiatum of hippocampal CA3 and CA1 with predominant innervation of the ventral hippocampus (Pikkarainen et al., 1999). Thus, the BLA-dorsal hippocampus signal transmission most likely follows an indirect polysynaptic route via the ventral hippocampus (Amaral and Witter, 1989). Lesions of the longitudinal hippocampal pathways demonstrated the functional significance of ventro-dorsal projections in spatial memory formation (Steffenach et al., 2002). The ventral hippocampus is also known to mediate contextual conditioning where ventral hippocampal lesions disrupt contextual freezing (Maren and Holt, 2004; Orsini et al., 2011; Kim and Cho, 2017; but see Huff et al., 2016). Furthermore, in vivo studies have shown that cells in the ventral region provide contextual information (Komorowski et al., 2013) and that ventral cells respond to odors much more strongly than dorsal cells (Keinath et al., 2014). This line of research reveals the role of the ventral hippocampus in fear and contextual conditioning and suggests that aversive signals may propagate across the hippocampal longitudinal axis towards the dorsal hippocampus that mediates spatial learning. We found that BLA photostimulation triggered a potent synchronization response in the dorsal hippocampal oscillations after light pulse delivery and increased power in the 5- to 8-Hz range. The firing of the hippocampal neurons also changed, with an increased extrafield spiking 100 ms after photostimulation, although we did not find hippocampal spikes that were directly entrained by the light pulses. Our electrophysiological results are indicative of a functional relationship between the amygdala and dorsal hippocampus; however, our histological data were insufficient to conclude whether this effect was mediated by the major indirect ventral projections or by sparse direct dorsal projections. Therefore, we consider that the indirect ventral pathway is the most likely anatomical route that mediates the observed amygdalo-hippocampal signaling.

We found that BLA-induced field remapping resembled the place field plasticity patterns after an aversive experience (TMT exposure). This finding should be considered in the context of two possible conditions, as follows: (1) the remapping patterns may have occurred not only as a result of the TMT or ChR2 protocol, but also due to the conflict between rewarding and aversive stimuli, and (2) the plasticity of dorsal hippocampal place cells may be mediated via different indirect pathways arising from the BLA. We propose that this pattern of field reconfiguration serves as a universal mechanism for the generation of multiple context-dependent representations by different salient stimuli, where the animal’s behavior is guided by the contextual valence of previous experience.

## Synthesis

Reviewing Editor: Liset Menendez de la Prida, Instituto Cajal CSIC Decisions are customarily a result of the Reviewing Editor and the peer reviewers coming together and discussing their recommendations until a consensus is reached. When revisions are invited, a fact-based synthesis statement explaining their decision and outlining what is needed to prepare a revision will be listed below.

Your ms was reviewed again and we feel the most critical technical issues are properly addressed. There is however the feeling that possibly as a result of the long review process the ms narrative reads a bit difficult. Somehow, results are presented so that it takes away from their novelty. We feel the ms will benefit from some editing. The problem is not ust grammatical, but the flow of ideas.

Here are some examples:

Lines 3-4: I would write : Here we report that rimethylthiazoline (TMT), an innately aversive odor, produces a reconfiguration of hippocampal representations.

Lines 608-625: The authors discuss the potential effect of reward as I suggested. Then they bring back the feeding zones out of the blue on lines 648-651 “Elimination of the feeding zones from the data  analyses would have excluded spikes from intra- or extra-field activity, affecting the reliability of the place field center of mass calculation and the accuracy of field plasticity  identification” . Topics should be organized within the same paragraph. Moreover, the previous sentence undermines the interpretation of the results. This should be eliminated since now the authors acknowledge that the feeders may play a role in the results.

Section “Amygdala mediates aversion-induced shift of the place field center of mass ”: it should be subdivided into paragraphs.

Please, check your ms throughout. Some entences in the abstract do not read well, paragraphs in the discussion are extremely long, and topics are intermixed. We will the value of your ms will be highlighted if you seek the help of an editor.

## Author Response

Synthesis Statement for Author (Required):

Your ms was reviewed again and we feel the most critical technical issues are properly addressed. There is however the feeling that possibly as a result of the long review process the ms narrative reads a bit difficult. Somehow, results are presented so that it takes away from their novelty. We feel the ms will benefit from some editing. The problem is not just grammatical, but the flow of ideas. Here are some examples:

Lines 3-4: I would write: Here we report that rimethylthiazoline (TMT), an innately aversive odor, produces a reconfiguration of hippocampal representations.

This sentence is now edited as suggested by the referee. With the assistance of English native speakers we have now improved the fluency of the presented ideas.

Lines 608-625: The authors discuss the potential effect of reward as I suggested. Then they bring back the feeding zones out of the blue on lines 648-651 “Elimination of the feeding zones from the data

analyses would have excluded spikes from intra- or extra-field activity, affecting the reliability of the place field center of mass calculation and the accuracy of field plasticity identification”. Topics should be organized within the same paragraph. Moreover, the previous sentence undermines the interpretation of the results. This should be eliminated since now the authors acknowledge that the feeders may play a role in the results. Section “Amygdala mediates aversion-induced shift of the place field center of mass ”: it should be subdivided into paragraphs.

The Discussion section is now revised as suggested by the referee. The disapproved by the referee sentence is now eliminated. We removed the following text from this paragraph: “The non-ChR2 zone included non-ChR2 arms and the feeding zones. Elimination of the feeding zones from the data

analyses would have excluded spikes from intra- or extra-field activity, affecting the reliability of the place field center of mass calculation and the accuracy of field plasticity identification”. Following the referee's advice we have now subdivided the section previously titled “Amygdala mediates aversion-induced shift of the place field center of mass” into two paragraphs titled: “Amygdala activation can shift the place field center of mass” and “Amygdala and hippocampus together mediate the acquisition of fear memory”.

Please, check your ms throughout. Some sentences in the abstract do not read well, paragraphs in the discussion are extremely long, and topics are intermixed. We will the value of your ms will be highlighted if you seek the help of an editor.

We have now edited some sentences to improve their comprehension (highlighted in bold font). We believe that the article is sufficiently edited to present clearly the novelty of our data. We thank the referee for the suggestions, which notably improved the scientific value of our work.
