# Supplementary material for: Extrafield Activity Shifts the Place Field Center of Mass to Encode Aversive Experience
Source: eNeuro. 2019 Mar 22;6(2):ENEURO.0423-17.2019. doi: 10.1523/ENEURO.0423-17.2019 (PMC6437659; doi:10.1523/ENEURO.0423-17.2019)
Supplement: Extended Data Figure 4-1 — Extrafield TMT spiking ratio and ΔCOMa of the place cells’ spikes in TMT arms. Download Figure 4-1, DOCX file. [file enu002192885so1.docx]

Figure 4-1. Extrafield TMT spiking ratio and ΔCOMa of the place cells’ spikes in TMT arms:

| Cell# | TMT Mean ratio | TMT Peak ratio | ΔCOMa | Cell# | TMT Mean ratio | TMT Peak ratio | ΔCOMa |
| --- | --- | --- | --- | --- | --- | --- | --- |
| 1 | 0.5 | 0.35 | 26.98 | 28 | 0.8 | 0.8 | 2.95 |
| 2 | 0.82 | 1.1 | 9.53 | 29 | 0.96 | 0.96 | 3.48 |
| 3 | 0.23 | 0.16 | 9.34 | 30 | 0.43 | 0.74 | 28.43 |
| 4 | 0.43 | 0.43 | 21.04 | 31 | 0.11 | 0.17 | 12.86 |
| 5 | 1 | 1 | 8.29 | 32 | 0.47 | 0.63 | 12.75 |
| 6 | 1.05 | 1.5 | 0.94 | 33 | 0.43 | 0.46 | 28.08 |
| 7 | 2 | 2.9 | 4.74 | 34 | 0.72 | 0.81 | 1.92 |
| 8 | 0.07 | 0.04 | 20.79 | 35 | 0.33 | 0.11 | 24.66 |
| 9 | 1 | 1.04 | 20.95 | 36 | 1.04 | 0.7 | 16.06 |
| 10 | 1.25 | 2.25 | 0.24 | 37 | 0.39 | 0.44 | 15.56 |
| 11 | 1.92 | 2.84 | 2.32 | 38 | 0.31 | 0.65 | 25.67 |
| 12 | 0.51 | 0.86 | 6.23 | 39 | 1.03 | 0.76 | 10 |
| 13 | 0.41 | 0.55 | 3.06 | 40 | 0.12 | 0.09 | 57.67 |
| 14 | 0.61 | 1.09 | 3.34 | 41 | 0.51 | 0.26 | 4.81 |
| 15 | 1 | 0.75 | 4.41 | 42 | 0.2 | 0.34 | 7.44 |
| 16 | 0.17 | 0.19 | 44.15 | 43 | 0.08 | 0.31 | 11.6 |
| 17 | 0.25 | 0.21 | 4.62 | 44 | 0.57 | 0.55 | 11.82 |
| 18 | 0.17 | 0.11 | 54.68 | 45 | 0.38 | 0.54 | 7.18 |
| 19 | 1.43 | 1.18 | 16.44 | 46 | 0.51 | 0.65 | 7.93 |
| 20 | 0.38 | 0.41 | 23.29 | 47 | 0.66 | 0.82 | 27.76 |
| 21 | 2.08 | 2.52 | 2.11 | 48 | 0.89 | 0.94 | 4.5 |
| 22 | 1.57 | 2.5 | 2.25 | 49 | 1.21 | 2.87 | 4.34 |
| 23 | 2.36 | 2.99 | 2.99 | 50 | 0.37 | 0.19 | 3.51 |
| 24 | 0.77 | 0.86 | 9.59 | 51 | 0.24 | 0.1 | 8.61 |
| 25 | 0.97 | 1.01 | 2.06 | 52 | 0.07 | 0.09 | 24.2 |
| 26 | 0.32 | 0.17 | 9.18 | 53 | 0.76 | 1.42 | 2.75 |
| 27 | 0.85 | 1.2 | 1.04 | 54 | 0.87 | 0.58 | 0.58 |
