# Supplementary material for: Extrafield Activity Shifts the Place Field Center of Mass to Encode Aversive Experience
Source: eNeuro. 2019 Mar 22;6(2):ENEURO.0423-17.2019. doi: 10.1523/ENEURO.0423-17.2019 (PMC6437659; doi:10.1523/ENEURO.0423-17.2019)
Supplement: Extended Data Figure 4-2 — Intrafield TMT spiking ratio and ΔCOMa of the place cells’ spikes in TMT arms. Download Figure 4-2, DOCX file. [file enu002192885so2.docx]

Figure 4-2. Intrafield TMT spiking ratio and ΔCOMa of the place cells’ spikes in TMT arms:

| Cell# | TMT Mean ratio | TMT Peak ratio | ΔCOMa | Cell# | TMT Mean ratio | TMT Peak ratio | ΔCOMa |
| --- | --- | --- | --- | --- | --- | --- | --- |
| 1 | 0.72 | 0.79 | 9.15 | 27 | 1.2 | 1.59 | 5.41 |
| 2 | 0.52 | 0.51 | 1.22 | 28 | 0.79 | 1.91 | 8.02 |
| 3 | 2.67 | 1.33 | 6.58 | 29 | 0.69 | 1.17 | 1.18 |
| 4 | 0.69 | 0.5 | 17.57 | 30 | 0.52 | 1.14 | 3.37 |
| 5 | 0.97 | 1.25 | 2.22 | 31 | 0.62 | 1.2 | 2.66 |
| 6 | 0.35 | 0.41 | 3.85 | 32 | 0.35 | 0.6 | 1.17 |
| 7 | 0.88 | 0.88 | 17.98 | 33 | 1.52 | 2.89 | 13.95 |
| 8 | 3.48 | 4.44 | 1.54 | 34 | 0.09 | 0.08 | 1.79 |
| 9 | 2.05 | 2.99 | 0.15 | 35 | 3.4 | 3.4 | 14.36 |
| 10 | 2.33 | 3.46 | 5.07 | 36 | 1.85 | 2.5 | 1.85 |
| 11 | 0.52 | 0.44 | 13.12 | 37 | 1 | 1.57 | 1.37 |
| 12 | 2.83 | 1.5 | 2.42 | 38 | 0.38 | 0.4 | 17.98 |
| 13 | 0.65 | 0.49 | 1.5 | 39 | 0.33 | 0.6 | 6.41 |
| 14 | 0.75 | 0.75 | 9.35 | 40 | 0.29 | 0.5 | 19.19 |
| 15 | 2.26 | 3.61 | 9.96 | 41 | 1.56 | 1.89 | 5.86 |
| 16 | 0.63 | 0.48 | 3.72 | 42 | 0.78 | 0.89 | 17.52 |
| 17 | 0.58 | 0.67 | 8.65 | 43 | 1.89 | 1.08 | 5.57 |
| 18 | 1.6 | 2.43 | 3.96 | 44 | 0.29 | 0.33 | 0.46 |
| 19 | 1.09 | 3.34 | 0.01 | 45 | 1.38 | 3.97 | 1.39 |
| 20 | 3.31 | 7.32 | 13.13 | 46 | 0.36 | 0.54 | 0.36 |
| 21 | 3.5 | 7.26 | 5.86 | 47 | 0.41 | 0.44 | 4.83 |
| 22 | 2.79 | 3.97 | 5.37 | 48 | 2.58 | 3.19 | 7.32 |
| 23 | 1.4 | 2.58 | 13.5 | 49 | 2.06 | 1.21 | 20.56 |
| 24 | 0.71 | 0.28 | 4.07 | 50 | 0.41 | 0.67 | 14.42 |
| 25 | 2.74 | 5 | 0.91 | 51 | 2.18 | 4.79 | 6.15 |
| 26 | 2.2 | 3.2 | 17.11 | 52 | 0.61 | 0.7 | 12.02 |
