# Supplementary material for: Extrafield Activity Shifts the Place Field Center of Mass to Encode Aversive Experience
Source: eNeuro. 2019 Mar 22;6(2):ENEURO.0423-17.2019. doi: 10.1523/ENEURO.0423-17.2019 (PMC6437659; doi:10.1523/ENEURO.0423-17.2019)
Supplement: Extended Data Figure 4-3 — Intrafield TMT spiking ratio and ΔCOMa of the place cells’ spikes in the non-TMT zone. Download Figure 4-3, DOCX file. [file enu002192885so3.docx]

Figure 4-3. Intrafield TMT spiking ratio and ΔCOMa of the place cells’ spikes in non-TMT zone:

| Cell# | TMT Mean ratio | TMT Peak ratio | ΔCOMa | Cell# | TMT Mean ratio | TMT Peak ratio | ΔCOMa |
| --- | --- | --- | --- | --- | --- | --- | --- |
| 1 | 3.13 | 2.68 | 26.98 | 29 | 0.23 | 0.22 | 2.95 |
| 2 | 1.4 | 0.64 | 9.53 | 30 | 0.27 | 0.2 | 3.48 |
| 3 | 2.1 | 2.78 | 9.34 | 31 | 0.38 | 0.42 | 28.43 |
| 4 | 1.07 | 1.35 | 26.03 | 32 | 1.35 | 1.44 | 12.86 |
| 5 | 3.33 | 2.35 | 21.04 | 33 | 0.69 | 1.87 | 12.75 |
| 6 | 0.67 | 1.45 | 0.94 | 34 | 0.41 | 0.33 | 28.08 |
| 7 | 1.39 | 2 | 4.74 | 35 | 0.58 | 1 | 1.92 |
| 8 | 1.15 | 0.48 | 20.79 | 36 | 0.58 | 0.27 | 24.66 |
| 9 | 0.68 | 0.67 | 10.77 | 37 | 0.9 | 0.47 | 16.06 |
| 10 | 0.77 | 0.69 | 3.18 | 38 | 0.78 | 0.3 | 15.56 |
| 11 | 0.71 | 0.38 | 16.28 | 39 | 1.32 | 1.12 | 25.67 |
| 12 | 1.25 | 0.65 | 2.32 | 40 | 0.2 | 0.08 | 10 |
| 13 | 0.48 | 0.82 | 6.23 | 41 | 0.1 | 0.09 | 57.67 |
| 14 | 0.4 | 0.62 | 3.06 | 42 | 0.19 | 0.08 | 4.81 |
| 15 | 0.6 | 0.71 | 3.34 | 43 | 0.25 | 0.17 | 7.44 |
| 16 | 3.57 | 2.63 | 4.41 | 44 | 0.03 | 0.02 | 11.6 |
| 17 | 0.85 | 0.46 | 44.15 | 45 | 1.07 | 0.71 | 11.82 |
| 18 | 0.42 | 0.36 | 4.62 | 46 | 1.21 | 0.4 | 7.18 |
| 19 | 0.96 | 0.79 | 54.68 | 47 | 0.22 | 0.22 | 7.93 |
| 20 | 0.66 | 0.63 | 16.44 | 48 | 0.88 | 1.09 | 27.76 |
| 21 | 1.24 | 0.67 | 23.29 | 49 | 1.04 | 1.5 | 4.5 |
| 22 | 2.54 | 2.5 | 2.11 | 50 | 1.92 | 2.23 | 4.34 |
| 23 | 1.79 | 1.33 | 1.39 | 51 | 0.44 | 0.44 | 3.51 |
| 24 | 3.38 | 4.18 | 3.31 | 52 | 0.6 | 0.54 | 8.61 |
| 25 | 1.02 | 0.68 | 9.59 | 53 | 4.2 | 4.17 | 24.2 |
| 26 | 2.8 | 5.26 | 2.06 | 54 | 0.45 | 0.25 | 2.75 |
| 27 | 0.4 | 0.12 | 9.18 | 55 | 1.38 | 0.82 | 1.95 |
| 28 | 0.72 | 0.84 | 1.04 | 56 |  |  |  |
