# Supplementary material for: Extrafield Activity Shifts the Place Field Center of Mass to Encode Aversive Experience
Source: eNeuro. 2019 Mar 22;6(2):ENEURO.0423-17.2019. doi: 10.1523/ENEURO.0423-17.2019 (PMC6437659; doi:10.1523/ENEURO.0423-17.2019)
Supplement: Extended Data Figure 4-4 — Extrafield TMT spiking ratio and ΔCOMa of the place cells’ spikes in the non-TMT zone. Download Figure 4-4, DOCX file. [file enu002192885so4.docx]

Figure 4-4. Extrafield TMT spiking ratio and ΔCOMa of the place cells’ spikes in non-TMT zone:

| Cell# | TMT Mean ratio | TMT Peak ratio | ΔCOMa | Cell# | TMT Mean ratio | TMT Peak ratio | ΔCOMa |
| --- | --- | --- | --- | --- | --- | --- | --- |
| 1 | 1.55 | 1.87 | 9.15 | 27 | 0.26 | 0.24 | 8.02 |
| 2 | 0.81 | 1.17 | 1.22 | 28 | 0.16 | 0.09 | 1.18 |
| 3 | 2.63 | 1.67 | 6.58 | 29 | 0.39 | 0.15 | 3.37 |
| 4 | 2 | 3.25 | 2.22 | 30 | 0.96 | 2.82 | 2.66 |
| 5 | 0.44 | 0.33 | 3.85 | 31 | 4.62 | 5.39 | 1.17 |
| 6 | 3.33 | 6.06 | 17.98 | 32 | 0.86 | 1.67 | 13.95 |
| 7 | 0.43 | 0.33 | 1.54 | 33 | 0.5 | 0.41 | 1.79 |
| 8 | 1.25 | 2.09 | 0.15 | 34 | 0.46 | 1.25 | 14.36 |
| 9 | 1.12 | 1.25 | 5.07 | 35 | 1.18 | 1.13 | 1.85 |
| 10 | 0.47 | 0.5 | 13.12 | 36 | 1.14 | 1.14 | 1.37 |
| 11 | 0.86 | 0.91 | 2.42 | 37 | 0.59 | 0.4 | 17.98 |
| 12 | 0.83 | 0.75 | 1.5 | 38 | 0.65 | 0.4 | 6.41 |
| 13 | 2.33 | 1.27 | 9.35 | 39 | 0.35 | 0.13 | 19.19 |
| 14 | 0.88 | 1.5 | 9.96 | 40 | 0.22 | 0.12 | 5.86 |
| 15 | 2.93 | 5.56 | 3.72 | 41 | 0.18 | 0.06 | 17.52 |
| 16 | 0.36 | 1 | 8.65 | 42 | 0.59 | 0.5 | 5.57 |
| 17 | 1.06 | 1.03 | 3.96 | 43 | 1.1 | 1.88 | 0.46 |
| 18 | 0.21 | 0.08 | 0.01 | 44 | 0.26 | 0.22 | 1.39 |
| 19 | 0.51 | 0.2 | 13.13 | 45 | 1.02 | 0.92 | 0.36 |
| 20 | 2.41 | 4 | 5.86 | 46 | 0.44 | 0.33 | 4.83 |
| 21 | 0.84 | 1 | 5.37 | 47 | 0.35 | 0.22 | 7.32 |
| 22 | 1.67 | 1 | 13.5 | 48 | 0.94 | 0.94 | 1.77 |
| 23 | 3.12 | 2.2 | 4.07 | 49 | 2.82 | 3.56 | 20.56 |
| 24 | 1.98 | 1.98 | 0.91 | 50 | 1.99 | 4.39 | 14.42 |
| 25 | 0.58 | 0.22 | 17.11 | 51 | 1.82 | 3.05 | 6.15 |
| 26 | 1.05 | 1 | 5.41 | 52 | 1.18 | 0.86 | 12.02 |
