# Supplementary material for: Extrafield Activity Shifts the Place Field Center of Mass to Encode Aversive Experience
Source: eNeuro. 2019 Mar 22;6(2):ENEURO.0423-17.2019. doi: 10.1523/ENEURO.0423-17.2019 (PMC6437659; doi:10.1523/ENEURO.0423-17.2019)
Supplement: Extended Data Figure 5-1 — Unidirectional ethanol spiking comparison and ΔCOM for clockwise fields. Download Figure 5-1, DOCX file. [file enu002192885so5.docx]

Figure 5-1. Unidirectional ethanol spiking comparison and ΔCOM for clockwise fields:

| Cell# | Mean rate | Peak rate | ΔCOM | Cell# | Mean rate | Peak rate | ΔCOM |
| --- | --- | --- | --- | --- | --- | --- | --- |
| 1 | 0.020 | 0.013 | 7.00 | 41 | 0.123 | 0.103 | 0.00 |
| 2 | 0.660 | 0.516 | 6.71 | 42 | -0.049 | -0.206 | 16.28 |
| 3 | 0.141 | 0.100 | 0.00 | 43 | 0.440 | 0.498 | 6.00 |
| 4 | 0.167 | 0.130 | 0.00 | 44 | -0.024 | 0.144 | 5.00 |
| 5 | -0.368 | -0.400 | 0.00 | 45 | 0.037 | 0.116 | 3.00 |
| 6 | -0.137 | -0.143 | 0.00 | 46 | -0.356 | -0.576 | 6.71 |
| 7 | 0.200 | 0.241 | 4.00 |  |  |  |  |
| 8 | -0.289 | -0.204 | 4.00 |  |  |  |  |
| 9 | -0.195 | -0.425 | 7.00 |  |  |  |  |
| 10 | 0.134 | 0.355 | 7.00 |  |  |  |  |
| 11 | -0.238 | -0.313 | 3.00 |  |  |  |  |
| 12 | -0.210 | -0.053 | 13.00 |  |  |  |  |
| 13 | 0.294 | 0.227 | 6.00 |  |  |  |  |
| 14 | 0.535 | 0.107 | 4.24 |  |  |  |  |
| 15 | 0.150 | 0.220 | 2.00 |  |  |  |  |
| 16 | 0.497 | 0.678 | 7.62 |  |  |  |  |
| 17 | -0.309 | -0.357 | 0.00 |  |  |  |  |
| 18 | -0.268 | 0.506 | 10.44 |  |  |  |  |
| 19 | -0.421 | -0.425 | 0.00 |  |  |  |  |
| 20 | -0.551 | -0.634 | 5.00 |  |  |  |  |
| 21 | -0.260 | -0.507 | 4.00 |  |  |  |  |
| 22 | 0.380 | 0.434 | 3.00 |  |  |  |  |
| 23 | 0.217 | 0.350 | 3.00 |  |  |  |  |
| 24 | -0.032 | -0.044 | 6.71 |  |  |  |  |
| 25 | 0.000 | 0.038 | 0.00 |  |  |  |  |
| 26 | 0.013 | -0.095 | 10.00 |  |  |  |  |
| 27 | 0.343 | 0.313 | 4.24 |  |  |  |  |
| 28 | 0.080 | -0.067 | 9.22 |  |  |  |  |
| 29 | -0.135 | -0.168 | 3.00 |  |  |  |  |
| 30 | 0.088 | 0.078 | 5.00 |  |  |  |  |
| 31 | -0.147 | -0.240 | 5.00 |  |  |  |  |
| 32 | 0.054 | -0.036 | 7.00 |  |  |  |  |
| 33 | -0.070 | 0.053 | 16.00 |  |  |  |  |
| 34 | -0.233 | -0.104 | 4.24 |  |  |  |  |
| 35 | -0.154 | 0.007 | 7.21 |  |  |  |  |
| 36 | 0.040 | 0.041 | 10.44 |  |  |  |  |
| 37 | 0.058 | -0.109 | 0.00 |  |  |  |  |
| 38 | 0.250 | 0.402 | 7.00 |  |  |  |  |
| 39 | 0.061 | 0.026 | 3.00 |  |  |  |  |
| 40 | 0.025 | 0.089 | 3.00 |  |  |  |  |
