# Supplementary material for: Extrafield Activity Shifts the Place Field Center of Mass to Encode Aversive Experience
Source: eNeuro. 2019 Mar 22;6(2):ENEURO.0423-17.2019. doi: 10.1523/ENEURO.0423-17.2019 (PMC6437659; doi:10.1523/ENEURO.0423-17.2019)
Supplement: Extended Data Figure 5-2 — Unidirectional ethanol spiking comparison and ΔCOM for counter-clockwise fields. Download Figure 5-2, DOCX file. [file enu002192885so6.docx]

Figure 5-2. Unidirectional ethanol spiking comparison and ΔCOM, counter-clockwise fields:

| Cell# | Mean rate | Peak rate | ΔCOM | Cell# | Mean rate | Peak rate | ΔCOM |
| --- | --- | --- | --- | --- | --- | --- | --- |
| 1 | 0.208 | 0.046 | 6.00 |  |  |  |  |
| 2 | -0.241 | -0.320 | 6.00 |  |  |  |  |
| 3 | 0.191 | 0.054 | 6.00 |  |  |  |  |
| 4 | -0.245 | -0.212 | 5.00 |  |  |  |  |
| 5 | -0.097 | 0.009 | 4.00 |  |  |  |  |
| 6 | 0.216 | 0.099 | 4.24 |  |  |  |  |
| 7 | 0.040 | 0.202 | 3.00 |  |  |  |  |
| 8 | -0.300 | -0.150 | 7.62 |  |  |  |  |
| 9 | 0.333 | 0.500 | 3.00 |  |  |  |  |
| 10 | -0.412 | -0.336 | 9.00 |  |  |  |  |
| 11 | -0.092 | -0.189 | 9.00 |  |  |  |  |
| 12 | 0.615 | 0.783 | 3.00 |  |  |  |  |
| 13 | 0.027 | -0.003 | 0.00 |  |  |  |  |
| 14 | -0.303 | -0.201 | 7.00 |  |  |  |  |
| 15 | 0.031 | 0.184 | 10.44 |  |  |  |  |
| 16 | 0.351 | 0.400 | 3.00 |  |  |  |  |
| 17 | -0.193 | 0.016 | 6.00 |  |  |  |  |
| 18 | 0.543 | 0.519 | 10.44 |  |  |  |  |
| 19 | 0.000 | -0.077 | 4.24 |  |  |  |  |
| 20 | 0.183 | 0.154 | 0.00 |  |  |  |  |
| 21 | 0.028 | 0.071 | 4.24 |  |  |  |  |
| 22 | 0.482 | 0.550 | 3.00 |  |  |  |  |
| 23 | 0.162 | 0.038 | 3.00 |  |  |  |  |
| 24 | 0.167 | 0.282 | 10.44 |  |  |  |  |
| 25 | 0.169 | 0.167 | 8.49 |  |  |  |  |
| 26 | -0.200 | -0.067 | 5.00 |  |  |  |  |
| 27 | -0.027 | -0.053 | 6.00 |  |  |  |  |
| 28 | -0.255 | -0.246 | 10.44 |  |  |  |  |
| 29 | 0.043 | 0.029 | 3.00 |  |  |  |  |
| 30 | -0.362 | -0.483 | 5.66 |  |  |  |  |
| 31 | -0.102 | 0.058 | 6.00 |  |  |  |  |
| 32 | -0.028 | -0.039 | 6.71 |  |  |  |  |
| 33 | -0.052 | -0.067 | 3.00 |  |  |  |  |
| 34 | -0.206 | -0.335 | 13.00 |  |  |  |  |
| 35 | 0.053 | -0.196 | 7.00 |  |  |  |  |
| 36 | 0.437 | 0.025 | 9.22 |  |  |  |  |
| 37 | -0.075 | -0.064 | 0.00 |  |  |  |  |
| 38 | 0.012 | -0.017 | 4.00 |  |  |  |  |
| 39 | -0.364 | -0.361 | 0.00 |  |  |  |  |
| 40 | -0.175 | -0.088 | 0.00 |  |  |  |  |
