# Supplementary material for: Extrafield Activity Shifts the Place Field Center of Mass to Encode Aversive Experience
Source: eNeuro. 2019 Mar 22;6(2):ENEURO.0423-17.2019. doi: 10.1523/ENEURO.0423-17.2019 (PMC6437659; doi:10.1523/ENEURO.0423-17.2019)
Supplement: Extended Data Figure 5-3 — Unidirectional TMT-SW spiking comparison and ΔCOM for clockwise fields. Download Figure 5-3, DOCX file. [file enu002192885so7.docx]

Figure 5-3. Unidirectional TMT-SW spiking comparison and ΔCOM for clockwise fields:

| Cell# | Mean rate | Peak rate | ΔCOM | Cell# | Mean rate | Peak rate | ΔCOM |
| --- | --- | --- | --- | --- | --- | --- | --- |
| 1 | -0.128 | -0.096 | 0.00 | 41 | 0.125 | 0.200 | 6.00 |
| 2 | 0.048 | 0.170 | 10.00 | 42 | 0.038 | 0.012 | 3.00 |
| 3 | -0.485 | -0.534 | 67.54 | 43 | -0.089 | -0.033 | 3.00 |
| 4 | -0.061 | 0.029 | 79.25 | 44 | -0.277 | -0.088 | 7.62 |
| 5 | 0.356 | 0.337 | 0.00 | 45 | 0.167 | 0.275 | 56.08 |
| 6 | 0.286 | 0.026 | 4.00 | 46 | 0.308 | 0.175 | 7.21 |
| 7 | 0.111 | 0.092 | 17.46 | 47 | 0.355 | 0.377 | 16.00 |
| 8 | 0.333 | 0.079 | 32.00 | 48 | 0.000 | -0.078 | 30.48 |
| 9 | -0.145 | -0.372 | 32.14 | 49 | -0.231 | -0.311 | 6.71 |
| 10 | -0.078 | 0.023 | 3.00 | 50 | 0.034 | -0.709 | 35.13 |
| 11 | 0.117 | -0.003 | 70.60 | 51 | 0.803 | 0.754 | 71.61 |
| 12 | -0.407 | -0.499 | 6.32 | 52 | -0.263 | -0.032 | 55.95 |
| 13 | 0.021 | 0.153 | 13.60 |  |  |  |  |
| 14 | 0.361 | 0.453 | 3.00 |  |  |  |  |
| 15 | -0.190 | -0.187 | 9.49 |  |  |  |  |
| 16 | 0.496 | 0.534 | 0.00 |  |  |  |  |
| 17 | 0.343 | 0.596 | 72.45 |  |  |  |  |
| 18 | -0.226 | -0.524 | 0.00 |  |  |  |  |
| 19 | -0.444 | -0.295 | 4.24 |  |  |  |  |
| 20 | 0.538 | 0.619 | 8.06 |  |  |  |  |
| 21 | 0.150 | 0.262 | 7.00 |  |  |  |  |
| 22 | 0.448 | 0.370 | 11.00 |  |  |  |  |
| 23 | 0.137 | 0.074 | 55.95 |  |  |  |  |
| 24 | 0.429 | 0.189 | 41.34 |  |  |  |  |
| 25 | -0.516 | -0.473 | 24.00 |  |  |  |  |
| 26 | -0.533 | -0.456 | 30.68 |  |  |  |  |
| 27 | 0.000 | 0.231 | 22.20 |  |  |  |  |
| 28 | -0.167 | 0.091 | 9.00 |  |  |  |  |
| 29 | -0.247 | -0.218 | 10.44 |  |  |  |  |
| 30 | 0.314 | 0.370 | 8.06 |  |  |  |  |
| 31 | -0.016 | 0.163 | 3.00 |  |  |  |  |
| 32 | -0.462 | -0.433 | 43.17 |  |  |  |  |
| 33 | -0.159 | -0.098 | 0.00 |  |  |  |  |
| 34 | 0.490 | 0.429 | 69.77 |  |  |  |  |
| 35 | -0.152 | 0.424 | 29.27 |  |  |  |  |
| 36 | 0.443 | 0.538 | 4.00 |  |  |  |  |
| 37 | -0.083 | -0.183 | 4.00 |  |  |  |  |
| 38 | 0.000 | 0.000 | 0.00 |  |  |  |  |
| 39 | -0.680 | -0.273 | 30.87 |  |  |  |  |
| 40 | -0.038 | 0.100 | 0.00 |  |  |  |  |
