# Supplementary material for: Extrafield Activity Shifts the Place Field Center of Mass to Encode Aversive Experience
Source: eNeuro. 2019 Mar 22;6(2):ENEURO.0423-17.2019. doi: 10.1523/ENEURO.0423-17.2019 (PMC6437659; doi:10.1523/ENEURO.0423-17.2019)
Supplement: Extended Data Figure 5-4 — Unidirectional TMT-SW spiking comparison and ΔCOM for counter-clockwise fields. Download Figure 5-4, DOCX file. [file enu002192885so8.docx]

Fig. 5-4. Unidirectional TMT-SW spiking comparison and ΔCOM, counter-clockwise fields:

| Cell# | Mean rate | Peak rate | ΔCOM | Cell# | Mean rate | Peak rate | ΔCOM |
| --- | --- | --- | --- | --- | --- | --- | --- |
| 1 | -0.471 | -0.478 | 4.00 | 40 | 0.135 | -0.216 | 55.00 |
| 2 | -0.247 | -0.288 | 0.00 | 41 | -0.165 | -0.424 | 61.03 |
| 3 | 0.407 | 0.444 | 76.85 | 42 | -0.745 | -0.740 | 32.25 |
| 4 | 0.081 | 0.297 | 0.00 | 43 | -0.214 | 0.081 | 70.60 |
| 5 | -0.263 | -0.351 | 78.24 | 44 | -0.200 | 0.077 | 9.00 |
| 6 | 0.864 | 0.922 | 20.40 | 45 | -0.589 | -0.712 | 55.80 |
| 7 | 0.778 | 0.741 | 0.00 | 46 | 0.135 | -0.216 | 55.00 |
| 8 | 0.368 | 0.000 | 35.00 |  |  |  |  |
| 9 | 0.544 | 0.717 | 16.00 |  |  |  |  |
| 10 | 0.231 | 0.403 | 5.00 |  |  |  |  |
| 11 | -0.205 | 0.072 | 5.39 |  |  |  |  |
| 12 | -0.391 | -0.259 | 0.00 |  |  |  |  |
| 13 | -0.277 | 0.000 | 69.86 |  |  |  |  |
| 14 | 0.105 | 0.207 | 0.00 |  |  |  |  |
| 15 | 0.300 | 0.034 | 0.00 |  |  |  |  |
| 16 | 0.143 | 0.042 | 29.15 |  |  |  |  |
| 17 | -0.341 | -0.427 | 6.00 |  |  |  |  |
| 18 | 0.302 | 0.229 | 64.85 |  |  |  |  |
| 19 | 0.037 | -0.024 | 75.29 |  |  |  |  |
| 20 | 0.448 | 0.267 | 41.44 |  |  |  |  |
| 21 | 0.250 | 0.416 | 9.00 |  |  |  |  |
| 22 | 0.121 | 0.194 | 0.00 |  |  |  |  |
| 23 | -0.095 | 0.000 | 16.28 |  |  |  |  |
| 24 | 0.091 | 0.302 | 76.55 |  |  |  |  |
| 25 | 0.000 | 0.167 | 8.06 |  |  |  |  |
| 26 | -0.135 | -0.081 | 0.00 |  |  |  |  |
| 27 | 0.118 | -0.114 | 7.62 |  |  |  |  |
| 28 | -0.364 | -0.354 | 9.90 |  |  |  |  |
| 29 | -0.357 | -0.538 | 54.92 |  |  |  |  |
| 30 | -0.487 | -0.200 | 7.62 |  |  |  |  |
| 31 | -0.051 | -0.087 | 4.24 |  |  |  |  |
| 32 | 0.143 | 0.156 | 19.00 |  |  |  |  |
| 33 | 0.128 | 0.231 | 11.00 |  |  |  |  |
| 34 | -0.420 | -0.495 | 3.00 |  |  |  |  |
| 35 | 0.204 | 0.178 | 7.00 |  |  |  |  |
| 36 | 0.014 | -0.109 | 10.44 |  |  |  |  |
| 37 | 0.942 | 0.859 | 64.85 |  |  |  |  |
| 38 | 0.518 | 0.479 | 3.00 |  |  |  |  |
| 39 | 0.191 | 0.172 | 10.44 |  |  |  |  |
| 40 | -0.508 | -0.653 | 0.00 |  |  |  |  |
