# Supplementary material for: Extrafield Activity Shifts the Place Field Center of Mass to Encode Aversive Experience
Source: eNeuro. 2019 Mar 22;6(2):ENEURO.0423-17.2019. doi: 10.1523/ENEURO.0423-17.2019 (PMC6437659; doi:10.1523/ENEURO.0423-17.2019)
Supplement: Extended Data Figure 5-5 — Unidirectional TMT-NE spiking comparison and ΔCOM for clockwise fields. Download Figure 5-5, DOCX file. [file enu002192885so9.docx]

Figure 5-5. Unidirectional TMT-NE spiking comparison and ΔCOM for clockwise fields:

| Cell# | Mean rate | Peak rate | ΔCOM | Cell# | Mean rate | Peak rate | ΔCOM |
| --- | --- | --- | --- | --- | --- | --- | --- |
| 1 | -0.295 | -0.308 | 10.00 |  |  |  |  |
| 2 | -0.613 | -0.820 | 42.11 |  |  |  |  |
| 3 | 0.476 | 0.500 | 55.54 |  |  |  |  |
| 4 | 0.333 | 0.219 | 7.21 |  |  |  |  |
| 5 | 0.692 | 0.610 | 99.70 |  |  |  |  |
| 6 | 0.141 | -0.018 | 3.00 |  |  |  |  |
| 7 | -0.143 | 0.000 | 7.00 |  |  |  |  |
| 8 | -0.375 | 0.120 | 10.77 |  |  |  |  |
| 9 | -0.057 | 0.098 | 4.00 |  |  |  |  |
| 10 | 0.619 | -0.667 | 71.47 |  |  |  |  |
| 11 | 0.155 | 0.468 | 3.00 |  |  |  |  |
| 12 | -0.046 | -0.137 | 16.49 |  |  |  |  |
| 13 | 0.125 | 0.185 | 16.49 |  |  |  |  |
| 14 | 0.246 | 0.301 | 6.71 |  |  |  |  |
| 15 | -0.406 | -0.468 | 58.08 |  |  |  |  |
| 16 | 0.360 | 0.172 | 19.24 |  |  |  |  |
| 17 | 0.623 | 0.621 | 22.20 |  |  |  |  |
| 18 | -0.202 | -0.033 | 6.71 |  |  |  |  |
| 19 | 0.667 | 0.762 | 7.62 |  |  |  |  |
| 20 | -0.370 | -0.467 | 41.88 |  |  |  |  |
| 21 | -0.786 | -0.746 | 73.00 |  |  |  |  |
| 22 | 0.094 | 0.316 | 10.44 |  |  |  |  |
| 23 | 0.000 | 0.000 | 36.12 |  |  |  |  |
| 24 | 0.340 | 0.411 | 25.00 |  |  |  |  |
| 25 | 0.042 | 0.000 | 4.24 |  |  |  |  |
| 26 | 0.145 | 0.272 | 4.00 |  |  |  |  |
| 27 | 0.107 | 0.084 | 3.00 |  |  |  |  |
| 28 | -0.447 | -0.634 | 13.34 |  |  |  |  |
| 29 | 0.669 | 0.667 | 3.00 |  |  |  |  |
| 30 | -0.006 | 0.000 | 6.71 |  |  |  |  |
| 31 | 0.040 | 0.103 | 83.74 |  |  |  |  |
| 32 | 0.216 | -0.064 | 12.00 |  |  |  |  |
| 33 | -0.274 | 0.077 | 16.28 |  |  |  |  |
|  |  |  |  |  |  |  |  |
|  |  |  |  |  |  |  |  |
|  |  |  |  |  |  |  |  |
|  |  |  |  |  |  |  |  |
|  |  |  |  |  |  |  |  |
|  |  |  |  |  |  |  |  |
|  |  |  |  |  |  |  |  |
