# Supplementary material for: Extrafield Activity Shifts the Place Field Center of Mass to Encode Aversive Experience
Source: eNeuro. 2019 Mar 22;6(2):ENEURO.0423-17.2019. doi: 10.1523/ENEURO.0423-17.2019 (PMC6437659; doi:10.1523/ENEURO.0423-17.2019)
Supplement: Extended Data Figure 5-6 — Unidirectional TMT-NE spiking comparison and ΔCOM for counter-clockwise fields. Download Figure 5-6, DOCX file. [file enu002192885so10.docx]

Fig. 5-6. Unidirectional TMT-NE spiking comparison and ΔCOM, counter-clockwise fields:

| Cell# | Mean rate | Peak rate | ΔCOM | Cell# | Mean rate | Peak rate | ΔCOM |
| --- | --- | --- | --- | --- | --- | --- | --- |
| 1 | 0.643 | 0.729 | 70.33 |  |  |  |  |
| 2 | -0.818 | -0.455 | 0.00 |  |  |  |  |
| 3 | -0.130 | 0.000 | 3.00 |  |  |  |  |
| 4 | 0.254 | 0.440 | 3.00 |  |  |  |  |
| 5 | 0.000 | 0.238 | 0.00 |  |  |  |  |
| 6 | 0.000 | -0.774 | 0.00 |  |  |  |  |
| 7 | 0.167 | 0.154 | 45.10 |  |  |  |  |
| 8 | -0.250 | -0.333 | 5.00 |  |  |  |  |
| 9 | -0.485 | -0.761 | 3.00 |  |  |  |  |
| 10 | 0.061 | 0.283 | 48.00 |  |  |  |  |
| 11 | 0.134 | 0.170 | 3.00 |  |  |  |  |
| 12 | -0.360 | -0.587 | 42.00 |  |  |  |  |
| 13 | 0.294 | 0.551 | 10.20 |  |  |  |  |
| 14 | 0.200 | 0.079 | 47.42 |  |  |  |  |
| 15 | -0.656 | -0.471 | 19.24 |  |  |  |  |
| 16 | 0.000 | 0.000 | 67.07 |  |  |  |  |
| 17 | 0.500 | 0.676 | 3.00 |  |  |  |  |
| 18 | 0.138 | 0.073 | 0.00 |  |  |  |  |
| 19 | -0.626 | -0.650 | 6.71 |  |  |  |  |
| 20 | 0.333 | 0.432 | 38.12 |  |  |  |  |
| 21 | -0.065 | -0.009 | 69.86 |  |  |  |  |
| 22 | 0.741 | 0.722 | 6.00 |  |  |  |  |
| 23 | 0.586 | 0.649 | 0.00 |  |  |  |  |
| 24 | 0.232 | 0.166 | 3.00 |  |  |  |  |
| 25 | 0.638 | 0.639 | 7.62 |  |  |  |  |
| 26 | 0.188 | -0.288 | 20.22 |  |  |  |  |
| 27 | 0.561 | 0.603 | 56.86 |  |  |  |  |
| 28 | -0.158 | -0.474 | 7.00 |  |  |  |  |
| 29 | 0.061 | 0.167 | 3.00 |  |  |  |  |
| 30 | -0.211 | -0.221 | 6.00 |  |  |  |  |
| 31 | 0.319 | 0.354 | 67.74 |  |  |  |  |
| 32 | -0.608 | -0.658 | 65.07 |  |  |  |  |
| 33 | 0.314 | 0.344 | 28.28 |  |  |  |  |
| 34 | -0.358 | -0.244 | 29.15 |  |  |  |  |
| 35 | 0.366 | 0.471 | 72.80 |  |  |  |  |
|  |  |  |  |  |  |  |  |
|  |  |  |  |  |  |  |  |
|  |  |  |  |  |  |  |  |
|  |  |  |  |  |  |  |  |
|  |  |  |  |  |  |  |  |
