# Supplementary material for: Extrafield Activity Shifts the Place Field Center of Mass to Encode Aversive Experience
Source: eNeuro. 2019 Mar 22;6(2):ENEURO.0423-17.2019. doi: 10.1523/ENEURO.0423-17.2019 (PMC6437659; doi:10.1523/ENEURO.0423-17.2019)
Supplement: Extended Data Figure 11-1 — Extrafield ChR2 spiking ratio and ΔCOMa of the place cells’ spikes in ChR2 arms. Download Figure 11-1, DOCX file. [file enu002192885so11.docx]

Figure 11-1. Extrafield ChR2 spiking ratio and ΔCOMa of the place cells’ spikes in ChR2 arms:

| Cell# | ChR2 Mean ratio | ChR2 Peak ratio | ΔCOMa | Cell# | ChR2 Mean ratio | ChR2 Peak ratio | ΔCOMa |
| --- | --- | --- | --- | --- | --- | --- | --- |
| 1 | 1.11 | 1.27 | 5.35 | 20 | 0.16 | 0.08 | 24.8 |
| 2 | 1.25 | 1.4 | 3.77 | 21 | 0.69 | 0.69 | 4.9 |
| 3 | 1.44 | 1.51 | 5.77 | 22 | 1.18 | 1.26 | 4.36 |
| 4 | 1.85 | 1.37 | 2.5 | 23 | 0.74 | 1.5 | 10.71 |
| 5 | 0.24 | 0.14 | 15.3 | 24 | 0.6 | 0.6 | 22.84 |
| 6 | 0.4 | 0.28 | 6.42 | 25 | 4.63 | 2.25 | 4.83 |
| 7 | 1.11 | 1.03 | 1.91 | 26 | 3.38 | 1.32 | 6.12 |
| 8 | 2.25 | 2.25 | 0.84 | 27 | 1.89 | 0.9 | 0.31 |
| 9 | 1.22 | 1.82 | 7 | 28 | 1.47 | 1.29 | 8.24 |
| 10 | 0.61 | 0.22 | 19.98 | 29 | 1.02 | 1.5 | 6.31 |
| 11 | 3.63 | 2.11 | 7.88 | 30 | 0.09 | 0.03 | 35.98 |
| 12 | 0.45 | 0.39 | 3.42 | 31 | 1.7 | 1.4 | 30.23 |
| 13 | 0.79 | 0.79 | 9.01 | 32 | 1.16 | 1.52 | 9.13 |
| 14 | 0.6 | 0.6 | 3.54 | 33 | 0.66 | 0.26 | 5.65 |
| 15 | 1.21 | 2.23 | 22.4 | 34 | 0.33 | 0.1 | 23.15 |
| 16 | 0.28 | 0.31 | 34.73 | 35 | 0.66 | 0.33 | 13.63 |
| 17 | 4.4 | 3.55 | 0.53 | 36 | 1.06 | 1.1 | 4.33 |
| 18 | 2 | 1.3 | 1.02 | 37 | 0.57 | 0.31 | 7.89 |
| 19 | 0.73 | 0.36 | 16.87 | 38 | 0.67 | 0.67 | 17.14 |
