# Supplementary material for: Extrafield Activity Shifts the Place Field Center of Mass to Encode Aversive Experience
Source: eNeuro. 2019 Mar 22;6(2):ENEURO.0423-17.2019. doi: 10.1523/ENEURO.0423-17.2019 (PMC6437659; doi:10.1523/ENEURO.0423-17.2019)
Supplement: Extended Data Figure 11-2 — Intrafield ChR2 spiking ratio and ΔCOMa of the place cells’ spikes in ChR2 arms. Download Figure 11-2, DOCX file. [file enu002192885so12.docx]

Figure 11-2. Intrafield ChR2 spiking ratio and ΔCOMa of the place cells’ spikes in ChR2 arms:

| Cell# | ChR2 Mean ratio | ChR2 Peak ratio | ΔCOMa | Cell# | ChR2 Mean ratio | ChR2 Peak ratio | ΔCOMa |
| --- | --- | --- | --- | --- | --- | --- | --- |
| 1 | 1 | 2.23 | 3.98 | 22 | 0.75 | 0.57 | 2.74 |
| 2 | 0.43 | 0.7 | 9.09 | 23 | 1.65 | 3 | 14.66 |
| 3 | 1.17 | 1.5 | 15.53 | 24 | 0.46 | 0.83 | 26.21 |
| 4 | 2.79 | 2.34 | 7.69 | 25 | 0.17 | 0.25 | 9.42 |
| 5 | 1.2 | 1.05 | 1.17 | 26 | 1.84 | 2 | 5.81 |
| 6 | 0.98 | 0.99 | 8.21 | 27 | 1.5 | 1 | 2.37 |
| 7 | 0.92 | 1.25 | 1.22 | 28 | 1.43 | 1.74 | 1.65 |
| 8 | 0.91 | 1.12 | 1.59 | 29 | 1.12 | 1.88 | 6.51 |
| 9 | 1.07 | 0.57 | 5.46 | 30 | 0.56 | 0.56 | 6.42 |
| 10 | 0.7 | 0.32 | 12.57 | 31 | 1.5 | 1.06 | 15.42 |
| 11 | 0.14 | 0.04 | 31.44 | 32 | 1.19 | 1 | 5.2 |
| 12 | 1.58 | 1.72 | 5.74 | 33 | 1.44 | 1.43 | 0.51 |
| 13 | 1.11 | 1.14 | 9.87 | 34 | 1.01 | 1.5 | 14.79 |
| 14 | 1.51 | 1 | 1.53 | 35 | 0.9 | 2 | 0.8 |
| 15 | 0.64 | 0.43 | 5.51 | 36 | 1.68 | 2 | 16.4 |
| 16 | 1.4 | 1.5 | 0.19 | 37 | 1.29 | 1.5 | 8.2 |
| 17 | 0.98 | 1.5 | 0.19 | 38 | 1.53 | 3.75 | 2.49 |
| 18 | 0.37 | 0.29 | 16.3 | 39 | 0.88 | 1 | 2.67 |
| 19 | 1.04 | 1.29 | 7.41 | 40 | 0.6 | 0.52 | 6.23 |
| 20 | 0.49 | 1.33 | 6 | 41 | 0.78 | 0.52 | 2.18 |
| 21 | 0.82 | 1.08 | 4.23 |  |  |  |  |
