# Supplementary material for: Extrafield Activity Shifts the Place Field Center of Mass to Encode Aversive Experience
Source: eNeuro. 2019 Mar 22;6(2):ENEURO.0423-17.2019. doi: 10.1523/ENEURO.0423-17.2019 (PMC6437659; doi:10.1523/ENEURO.0423-17.2019)
Supplement: Extended Data Figure 11-3 — Intrafield ChR2 spiking ratio and ΔCOMa of the place cells’ spikes in the non-ChR2 zone. Download Figure 11-3, DOCX file. [file enu002192885so13.docx]

Figure 11-3. Intrafield ChR2 spiking ratio and ΔCOMa of the place cells’ spikes in non-ChR2 zone:

| Cell# | ChR2 Mean ratio | ChR2 Peak ratio | ΔCOMa | Cell# | ChR2 Mean ratio | ChR2 Peak ratio | ΔCOMa |
| --- | --- | --- | --- | --- | --- | --- | --- |
| 1 | 0.87 | 1.32 | 5.35 | 26 | 2.93 | 3.5 | 37.11 |
| 2 | 0.55 | 0.56 | 3.77 | 27 | 2 | 3.21 | 4.83 |
| 3 | 0.5 | 0.23 | 8.31 | 28 | 0.93 | 1.21 | 6.12 |
| 4 | 0.58 | 0.66 | 5.77 | 29 | 1.94 | 2.33 | 0.31 |
| 5 | 2.74 | 4 | 2.5 | 30 | 0.79 | 0.92 | 8.51 |
| 6 | 1.35 | 1.27 | 15.3 | 31 | 0.62 | 0.29 | 14.46 |
| 7 | 1.45 | 2.33 | 6.42 | 32 | 1.64 | 1.5 | 8.55 |
| 8 | 0.99 | 0.95 | 1.91 | 33 | 1.01 | 0.56 | 9.03 |
| 9 | 1.75 | 1.98 | 0.84 | 34 | 1.12 | 1.35 | 8.24 |
| 10 | 1.07 | 0.93 | 23.71 | 35 | 0.6 | 0.52 | 6.31 |
| 11 | 0.88 | 1.15 | 19.98 | 36 | 1.54 | 1.95 | 35.98 |
| 12 | 2.46 | 4.63 | 7.88 | 37 | 3.03 | 4.72 | 30.23 |
| 13 | 0.51 | 0.38 | 3.42 | 38 | 0.39 | 0.46 | 9.13 |
| 14 | 4.18 | 2.63 | 9.01 | 39 | 1.18 | 1.27 | 5.65 |
| 15 | 0.78 | 0.5 | 3.54 | 40 | 0.5 | 0.67 | 23.15 |
| 16 | 0.77 | 0.67 | 22.4 | 41 | 0.53 | 0.43 | 4.06 |
| 17 | 1.34 | 1 | 34.73 | 42 | 0.42 | 0.33 | 13.63 |
| 18 | 0.96 | 2.06 | 0.53 | 43 | 1.06 | 1.11 | 35.65 |
| 19 | 1.19 | 1 | 1.02 | 44 | 0.93 | 0.87 | 4.36 |
| 20 | 1.91 | 0.8 | 16.87 | 45 | 2.63 | 3 | 4.33 |
| 21 | 0.79 | 1 | 11.51 | 46 | 1.54 | 1.67 | 7.89 |
| 22 | 0.89 | 1.11 | 24.8 | 47 | 0.95 | 1 | 1.52 |
| 23 | 1.96 | 1.37 | 4.9 | 48 | 0.38 | 0.6 | 17.14 |
| 24 | 3.17 | 4.17 | 4.36 | 49 | 2.24 | 2.52 | 22.59 |
| 25 | 0.88 | 0.53 | 10.71 |  |  |  |  |
