# Supplementary material for: Extrafield Activity Shifts the Place Field Center of Mass to Encode Aversive Experience
Source: eNeuro. 2019 Mar 22;6(2):ENEURO.0423-17.2019. doi: 10.1523/ENEURO.0423-17.2019 (PMC6437659; doi:10.1523/ENEURO.0423-17.2019)
Supplement: Extended Data Figure 11-4 — Extrafield ChR2 spiking ratio and ΔCOMa of the place cells’ spikes in the non-ChR2 zone. Download Figure 11-4, DOCX file. [file enu002192885so14.docx]

Figure 11-4. Extrafield ChR2 spiking ratio and ΔCOMa of the place cells’ spikes in non-ChR2 zone:

| Cell# | ChR2 Mean ratio | ChR2 Peak ratio | ΔCOMa | Cell# | ChR2 Mean ratio | ChR2 Peak ratio | ΔCOMa |
| --- | --- | --- | --- | --- | --- | --- | --- |
| 1 | 0.79 | 1.44 | 3.98 | 22 | 1.11 | 0.58 | 2.74 |
| 2 | 1.43 | 3.01 | 9.09 | 23 | 0.1 | 0.09 | 14.66 |
| 3 | 0.31 | 0.42 | 15.53 | 24 | 1.37 | 3.7 | 26.21 |
| 4 | 1.28 | 1.49 | 7.69 | 25 | 1.02 | 1.11 | 9.42 |
| 5 | 1.07 | 1.25 | 1.17 | 26 | 1.07 | 1 | 5.81 |
| 6 | 1.41 | 0.71 | 8.21 | 27 | 1.27 | 1.22 | 2.37 |
| 7 | 1.05 | 0.5 | 1.22 | 28 | 0.86 | 0.56 | 1.65 |
| 8 | 0.65 | 1 | 1.59 | 29 | 0 | 0.01 | 6.51 |
| 9 | 1.21 | 1.24 | 5.46 | 30 | 0.64 | 0.64 | 6.42 |
| 10 | 1.96 | 1.5 | 12.57 | 31 | 1.39 | 1.25 | 15.42 |
| 11 | 1.69 | 1.73 | 31.44 | 32 | 0.69 | 0.79 | 5.2 |
| 12 | 1.35 | 1.09 | 5.74 | 33 | 0.75 | 0.78 | 0.51 |
| 13 | 3.03 | 3.13 | 9.87 | 34 | 2.8 | 2 | 14.79 |
| 14 | 2.18 | 5.65 | 1.53 | 35 | 3.22 | 5.77 | 0.8 |
| 15 | 0.89 | 0.83 | 5.51 | 36 | 0.79 | 0.77 | 12.48 |
| 16 | 1.2 | 0.56 | 0.19 | 37 | 0.77 | 0.6 | 16.4 |
| 17 | 0.67 | 0.45 | 0.19 | 38 | 0.92 | 1.25 | 8.2 |
| 18 | 1.9 | 1.93 | 16.3 | 39 | 1.45 | 1.68 | 2.49 |
| 19 | 2.81 | 2.75 | 7.41 | 40 | 2.29 | 5 | 2.67 |
| 20 | 0.92 | 1.11 | 6 | 41 | 1.53 | 2.7 | 6.23 |
| 21 | 0.51 | 0.85 | 4.23 | 42 | 0.38 | 0.25 | 2.18 |
