# Supplementary material for: Extrafield Activity Shifts the Place Field Center of Mass to Encode Aversive Experience
Source: eNeuro. 2019 Mar 22;6(2):ENEURO.0423-17.2019. doi: 10.1523/ENEURO.0423-17.2019 (PMC6437659; doi:10.1523/ENEURO.0423-17.2019)
Supplement: Extended Data Figure 12-1 — Unidirectional YFP spiking comparison and ΔCOM for clockwise fields. Download Figure 12-1, DOCX file. [file enu002192885so15.docx]

Figure 12-1. Unidirectional YFP spiking comparison and ΔCOM for clockwise fields:

| Cell# | Mean rate | Peak rate | ΔCOM | Cell# | Mean rate | Peak rate | ΔCOM |
| --- | --- | --- | --- | --- | --- | --- | --- |
| 1 | -0.259 | -0.220 | 5.00 | 41 | 0.184 | 0.286 | 0.00 |
| 2 | 0.036 | -0.055 | 0.00 | 42 | 0.074 | -0.024 | 4.00 |
| 3 | 0.089 | -0.035 | 10.00 | 43 | 0.033 | -0.028 | 3.00 |
| 4 | 0.444 | 0.399 | 10.44 | 44 | 0.512 | 0.031 | 21.47 |
| 5 | 0.052 | 0.000 | 5.10 |  |  |  |  |
| 6 | -0.250 | -0.206 | 3.00 |  |  |  |  |
| 7 | 0.091 | 0.023 | 4.24 |  |  |  |  |
| 8 | -0.674 | -0.802 | 39.46 |  |  |  |  |
| 9 | -0.169 | -0.305 | 3.00 |  |  |  |  |
| 10 | 0.190 | 0.140 | 3.00 |  |  |  |  |
| 11 | 0.059 | 0.132 | 0.00 |  |  |  |  |
| 12 | 0.228 | 0.161 | 9.00 |  |  |  |  |
| 13 | -0.271 | -0.309 | 4.24 |  |  |  |  |
| 14 | 0.143 | 0.200 | 0.00 |  |  |  |  |
| 15 | 0.262 | 0.280 | 10.00 |  |  |  |  |
| 16 | 0.240 | 0.357 | 3.00 |  |  |  |  |
| 17 | 0.429 | 0.400 | 4.24 |  |  |  |  |
| 18 | 0.680 | 0.558 | 3.00 |  |  |  |  |
| 19 | -0.263 | -0.256 | 0.00 |  |  |  |  |
| 20 | -0.077 | -0.079 | 6.00 |  |  |  |  |
| 21 | 0.035 | 0.065 | 3.00 |  |  |  |  |
| 22 | 0.182 | 0.016 | 6.00 |  |  |  |  |
| 23 | -0.025 | 0.358 | 18.11 |  |  |  |  |
| 24 | 0.333 | 0.488 | 6.00 |  |  |  |  |
| 25 | -0.433 | -0.299 | 0.00 |  |  |  |  |
| 26 | -0.068 | -0.179 | 0.00 |  |  |  |  |
| 27 | -0.333 | -0.482 | 4.00 |  |  |  |  |
| 28 | 0.537 | 0.571 | 7.00 |  |  |  |  |
| 29 | -0.048 | 0.116 | 10.44 |  |  |  |  |
| 30 | -0.058 | 0.124 | 5.00 |  |  |  |  |
| 31 | 0.147 | 0.143 | 0.00 |  |  |  |  |
| 32 | 0.163 | 0.000 | 8.06 |  |  |  |  |
| 33 | -0.321 | -0.579 | 16.28 |  |  |  |  |
| 34 | -0.077 | -0.103 | 4.00 |  |  |  |  |
| 35 | 0.114 | 0.329 | 0.00 |  |  |  |  |
| 36 | -0.421 | -0.195 | 0.00 |  |  |  |  |
| 37 | -0.440 | -0.319 | 0.00 |  |  |  |  |
| 38 | -0.306 | -0.263 | 7.00 |  |  |  |  |
| 39 | 0.150 | 0.282 | 3.00 |  |  |  |  |
| 40 | -0.042 | 0.034 | 0.00 |  |  |  |  |
