# Supplementary material for: Extrafield Activity Shifts the Place Field Center of Mass to Encode Aversive Experience
Source: eNeuro. 2019 Mar 22;6(2):ENEURO.0423-17.2019. doi: 10.1523/ENEURO.0423-17.2019 (PMC6437659; doi:10.1523/ENEURO.0423-17.2019)
Supplement: Extended Data Figure 12-2 — Unidirectional YFP spiking comparison and ΔCOM for counter-clockwise fields. Download Figure 12-2, DOCX file. [file enu002192885so16.docx]

Figure 12-2. Unidirectional YFP spiking comparison and ΔCOM, counter-clockwise fields:

| Cell# | Mean rate | Peak rate | ΔCOM | Cell# | Mean rate | Peak rate | ΔCOM |
| --- | --- | --- | --- | --- | --- | --- | --- |
| 1 | 0.037 | -0.055 | 0.00 |  |  |  |  |
| 2 | -0.281 | -0.257 | 7.00 |  |  |  |  |
| 3 | 0.167 | 0.026 | 14.32 |  |  |  |  |
| 4 | -0.383 | -0.283 | 13.00 |  |  |  |  |
| 5 | -0.010 | 0.036 | 2.83 |  |  |  |  |
| 6 | -0.005 | -0.102 | 3.00 |  |  |  |  |
| 7 | 0.059 | -0.152 | 13.34 |  |  |  |  |
| 8 | 0.259 | 0.261 | 3.00 |  |  |  |  |
| 9 | -0.008 | 0.099 | 10.00 |  |  |  |  |
| 10 | -0.009 | -0.161 | 3.00 |  |  |  |  |
| 11 | -0.086 | -0.013 | 0.00 |  |  |  |  |
| 12 | -0.023 | -0.011 | 0.00 |  |  |  |  |
| 13 | -0.309 | -0.091 | 6.00 |  |  |  |  |
| 14 | 0.200 | 0.390 | 0.00 |  |  |  |  |
| 15 | 0.054 | 0.131 | 6.71 |  |  |  |  |
| 16 | 0.350 | 0.284 | 3.00 |  |  |  |  |
| 17 | 0.319 | 0.293 | 0.00 |  |  |  |  |
| 18 | 0.035 | -0.153 | 3.00 |  |  |  |  |
| 19 | 0.118 | 0.010 | 13.60 |  |  |  |  |
| 20 | 0.000 | -0.298 | 3.00 |  |  |  |  |
| 21 | 0.100 | 0.539 | 4.24 |  |  |  |  |
| 22 | 0.405 | 0.432 | 4.00 |  |  |  |  |
| 23 | 0.188 | 0.091 | 16.16 |  |  |  |  |
| 24 | 0.371 | 0.290 | 0.00 |  |  |  |  |
| 25 | 0.232 | 0.359 | 4.00 |  |  |  |  |
| 26 | 0.100 | 0.231 | 0.00 |  |  |  |  |
| 27 | -0.143 | 0.083 | 0.00 |  |  |  |  |
| 28 | -0.475 | -0.405 | 3.00 |  |  |  |  |
| 29 | -0.256 | -0.473 | 4.00 |  |  |  |  |
| 30 | -0.161 | -0.029 | 3.00 |  |  |  |  |
| 31 | 0.125 | -0.032 | 4.00 |  |  |  |  |
| 32 | 0.486 | 0.379 | 7.00 |  |  |  |  |
| 33 | 0.231 | 0.434 | 5.00 |  |  |  |  |
| 34 | -0.770 | -0.815 | 3.00 |  |  |  |  |
| 35 | 0.208 | 0.304 | 0.00 |  |  |  |  |
| 36 | -0.684 | -0.600 | 6.00 |  |  |  |  |
| 37 | -0.660 | -0.597 | 10.44 |  |  |  |  |
| 38 | 0.302 | 0.333 | 3.00 |  |  |  |  |
| 39 | 0.102 | -0.004 | 0.00 |  |  |  |  |
| 40 | 0.028 | 0.333 | 5.66 |  |  |  |  |
