# Supplementary material for: Extrafield Activity Shifts the Place Field Center of Mass to Encode Aversive Experience
Source: eNeuro. 2019 Mar 22;6(2):ENEURO.0423-17.2019. doi: 10.1523/ENEURO.0423-17.2019 (PMC6437659; doi:10.1523/ENEURO.0423-17.2019)
Supplement: Extended Data Figure 12-3 — Unidirectional ChR2 spiking comparison and ΔCOM for clockwise fields. Download Figure 12-3, DOCX file. [file enu002192885so17.docx]

Figure 12-3. Unidirectional ChR2 spiking comparison and ΔCOM for clockwise fields:

| Cell# | Mean rate | Peak rate | ΔCOM | Cell# | Mean rate | Peak rate | ΔCOM |
| --- | --- | --- | --- | --- | --- | --- | --- |
| 1 | -0.437 | -0.472 | 4.24 | 41 | -0.500 | -0.694 | 45.10 |
| 2 | -0.080 | -0.004 | 0.00 | 42 | 0.534 | 0.277 | 26.00 |
| 3 | -0.179 | -0.282 | 6.00 | 43 | -0.183 | -0.011 | 66.85 |
| 4 | 0.388 | 0.415 | 74.06 | 44 | 0.531 | 0.613 | 10.00 |
| 5 | 0.355 | 0.083 | 39.05 | 45 | -0.564 | -0.500 | 4.00 |
| 6 | -0.355 | -0.304 | 6.71 | 46 | 0.000 | 0.000 | 4.00 |
| 7 | 0.205 | 0.053 | 7.00 | 47 | -0.200 | -0.160 | 5.10 |
| 8 | -0.143 | -0.190 | 9.43 | 48 | 0.500 | 0.465 | 86.27 |
| 9 | -0.472 | -0.541 | 75.80 | 49 | 0.300 | 0.347 | 60.88 |
| 10 | -0.263 | -0.288 | 3.00 | 50 | -0.392 | -0.471 | 95.46 |
| 11 | -0.185 | -0.083 | 7.62 | 51 | 0.667 | 0.799 | 73.41 |
| 12 | -0.250 | -0.244 | 3.00 | 52 | 0.046 | -0.059 | 5.00 |
| 13 | -0.212 | -0.324 | 3.00 | 53 | 1.000 | 0.972 | 63.06 |
| 14 | 0.096 | 0.014 | 7.00 | 54 | 0.767 | 0.836 | 7.62 |
| 15 | 0.288 | 0.307 | 10.00 | 55 | 0.116 | -0.269 | 55.54 |
| 16 | -0.438 | -0.446 | 22.00 | 56 | 0.464 | 0.454 | 4.00 |
| 17 | -0.565 | -0.721 | 3.00 | 57 | -0.212 | -0.194 | 6.71 |
| 18 | 0.600 | 0.475 | 7.00 | 58 | 0.042 | -0.045 | 50.54 |
| 19 | 0.733 | 0.857 | 48.00 | 59 | 0.230 | 0.308 | 6.00 |
| 20 | -0.080 | 0.183 | 4.24 | 60 | 0.012 | 0.173 | 3.00 |
| 21 | 0.500 | 0.545 | 4.24 | 61 | -0.438 | -0.603 | 6.00 |
| 22 | 0.088 | -0.074 | 65.92 | 62 | 0.167 | 0.200 | 57.01 |
| 23 | -0.866 | -0.761 | 48.09 | 63 | 0.182 | 0.422 | 2.00 |
| 24 | 0.000 | 0.353 | 13.00 | 64 | -0.539 | -0.581 | 5.00 |
| 25 | -0.571 | -0.619 | 76.55 | 65 | -0.366 | -0.449 | 3.00 |
| 26 | -0.121 | -0.021 | 3.00 | 66 | 0.231 | 0.280 | 74.06 |
| 27 | -0.023 | -0.031 | 55.80 | 67 | -0.171 | -0.261 | 0.00 |
| 28 | 0.457 | 0.371 | 0.00 | 68 | 0.150 | 0.117 | 32.14 |
| 29 | 0.500 | 0.783 | 64.20 | 69 | 0.479 | 0.466 | 71.06 |
| 30 | 0.234 | 0.327 | 41.23 | 70 | 0.156 | 0.113 | 0.00 |
| 31 | 0.311 | 0.117 | 51.04 | 71 | -0.143 | -0.039 | 2.00 |
| 32 | -0.259 | 0.240 | 22.00 | 72 | 0.136 | 0.168 | 42.15 |
| 33 | 0.247 | 0.232 | 10.00 | 73 | -0.040 | 0.131 | 16.28 |
| 34 | 0.008 | -0.188 | 3.16 | 74 | 0.116 | 0.379 | 26.00 |
| 35 | 0.547 | 0.485 | 0.00 | 75 | -0.280 | -0.149 | 7.00 |
| 36 | -0.186 | -0.091 | 56.86 | 76 | 0.811 | 0.867 | 18.03 |
| 37 | 0.164 | 0.311 | 10.44 | 77 | -0.035 | 0.003 | 4.24 |
| 38 | 0.726 | 0.795 | 43.91 | 78 | 0.481 | 0.534 | 0.00 |
| 39 | 0.185 | 0.228 | 3.00 | 79 | -0.098 | 0.018 | 2.24 |
| 40 | 0.176 | -0.250 | 2.24 | 80 | -0.474 | -0.450 | 7.00 |
