# Supplementary material for: Extrafield Activity Shifts the Place Field Center of Mass to Encode Aversive Experience
Source: eNeuro. 2019 Mar 22;6(2):ENEURO.0423-17.2019. doi: 10.1523/ENEURO.0423-17.2019 (PMC6437659; doi:10.1523/ENEURO.0423-17.2019)
Supplement: Extended Data Figure 12-4 — Unidirectional ChR2 spiking comparison and ΔCOM for counter-clockwise fields. Download Figure 12-4, DOCX file. [file enu002192885so18.docx]

Figure 12-4. Unidirectional ChR2 spiking comparison and ΔCOM, counter-clockwise fields:

| Cell# | Mean rate | Peak rate | ΔCOM | Cell# | Mean rate | Peak rate | ΔCOM |
| --- | --- | --- | --- | --- | --- | --- | --- |
| 1 | -0.083 | -0.049 | 75.69 | 41 | 0.622 | 0.762 | 3.00 |
| 2 | -0.143 | 0.036 | 3.00 | 42 | 0.138 | 0.129 | 20.00 |
| 3 | 0.607 | 0.790 | 19.00 | 43 | 0.000 | -0.011 | 4.00 |
| 4 | -0.286 | 0.148 | 73.41 | 44 | -0.211 | -0.200 | 3.00 |
| 5 | -0.295 | -0.175 | 66.85 | 45 | -0.570 | -0.465 | 68.01 |
| 6 | 0.226 | 0.273 | 3.00 | 46 | -0.014 | 0.127 | 0.00 |
| 7 | -0.780 | -0.728 | 10.30 | 47 | -0.207 | -0.383 | 8.06 |
| 8 | 0.425 | 0.506 | 10.00 | 48 | -0.639 | -0.538 | 32.14 |
| 9 | 0.154 | 0.368 | 76.55 | 49 | -0.316 | -0.092 | 3.00 |
| 10 | 0.132 | 0.000 | 4.24 | 50 | 0.000 | -0.370 | 3.00 |
| 11 | 0.333 | 0.111 | 32.14 | 51 | -0.091 | 0.184 | 51.00 |
| 12 | 0.683 | 0.632 | 5.66 | 52 | -0.176 | -0.205 | 7.07 |
| 13 | -0.352 | -0.564 | 36.12 | 53 | -0.539 | -0.581 | 5.00 |
| 14 | 0.000 | 0.157 | 3.00 | 54 | 0.323 | 0.406 | 4.00 |
| 15 | 0.158 | 0.000 | 86.37 | 55 | 0.306 | 0.359 | 10.00 |
| 16 | -0.111 | -0.290 | 28.28 | 56 | 0.254 | 0.262 | 7.00 |
| 17 | 0.600 | 0.345 | 48.85 | 57 | 0.376 | 0.517 | 5.66 |
| 18 | -0.754 | -0.822 | 42.58 | 58 | -0.522 | -0.597 | 9.49 |
| 19 | -0.100 | 0.043 | 70.29 | 59 | -0.288 | -0.470 | 13.00 |
| 20 | 0.800 | 0.917 | 16.28 | 60 | -0.731 | -0.670 | 52.09 |
| 21 | 0.200 | 0.273 | 23.00 | 61 | -0.412 | -0.226 | 16.00 |
| 22 | -0.739 | -0.766 | 42.11 | 62 | 0.455 | 0.443 | 0.00 |
| 23 | -0.125 | -0.184 | 77.52 | 63 | 0.321 | 0.085 | 4.00 |
| 24 | -0.004 | -0.009 | 12.00 | 64 | -0.143 | -0.178 | 3.00 |
| 25 | -0.162 | -0.081 | 10.44 | 65 | 0.003 | 0.005 | 4.00 |
| 26 | 0.392 | 0.429 | 7.00 | 66 | 0.250 | 0.467 | 84.17 |
| 27 | 0.897 | 0.965 | 60.17 | 67 | 0.301 | 0.144 | 4.24 |
| 28 | -0.120 | -0.035 | 6.00 | 68 | 0.223 | 0.348 | 35.51 |
| 29 | -0.548 | -0.554 | 9.49 | 69 | 0.004 | -0.019 | 3.00 |
| 30 | 0.690 | 0.575 | 8.06 | 70 | 0.429 | 0.435 | 23.00 |
| 31 | 0.425 | 0.451 | 9.00 | 71 | -0.053 | 0.000 | 3.00 |
| 32 | 0.254 | 0.311 | 65.97 | 72 | 0.742 | 0.841 | 63.64 |
| 33 | -0.484 | -0.621 | 10.00 | 73 | 0.118 | 0.206 | 4.00 |
| 34 | 0.923 | 0.913 | 16.28 | 74 | -0.257 | -0.095 | 76.55 |
| 35 | -0.009 | -0.214 | 1.41 |  |  |  |  |
| 36 | -0.123 | -0.136 | 6.71 |  |  |  |  |
| 37 | 0.362 | 0.492 | 9.85 |  |  |  |  |
| 38 | 0.297 | 0.242 | 9.85 |  |  |  |  |
| 39 | 0.429 | 0.497 | 29.61 |  |  |  |  |
| 40 | 0.636 | -0.056 | 25.02 |  |  |  |  |
